# Dermal Papilla Cell Proliferation of Phytochemicals Isolated from Chestnut Shells (*Castanea crenata*)

**DOI:** 10.3390/plants12051018

**Published:** 2023-02-23

**Authors:** SeonJu Park, Nahyun Choi, Le Nu Huyen Trang, Mira Oh, Youngse Oh, Jong-Hyuk Sung, Seung Hyun Kim

**Affiliations:** 1 Chuncheon Center, Korea Basic Science Institute (KBSI), Chuncheon 24341, Republic of Korea; 2 Epibiotech Co., Ltd., Incheon 21984, Republic of Korea; 3 Yonsei Institute of Pharmaceutical Sciences, College of Pharmacy, Yonsei University, Incheon 21983, Republic of Korea; 4 Research Group of Traditional Food, Korea Food Research Institute, Wanju-gun 55365, Republic of Korea

**Keywords:** *Castanea crenata* shell, Fagaceae, seco-*ent*-diterpenes, phenolics, dermal papilla cell proliferation

## Abstract

*Castanea crenata* (Fagaceae) is a species of chestnut tree that is endemic to the Republic of Korea and Japan. While its kernels are consumed, chestnut by-products such as shells and burs, which account for 10–15% of the total weight, are discarded as waste. Phytochemical and biological studies have been carried out to eliminate this waste and develop high-value products from its by-products. In this study, five new compounds (**1**–**2**, **6**–**8**) along with seven known compounds were isolated from the shell of *C. crenata*. This is the first study to report diterpenes from the shell of *C. crenata*. Comprehensive spectroscopic data including 1D, 2D NMR, and CD spectroscopy were used to determine the compound structures. All isolated compounds were examined for their ability to stimulate dermal papilla cell proliferation using a CCK-8 assay. In particular, 6β,7β,16α,17-Tetrahydroxy-*ent*-kauranoic acid, isopentyl-α-L-arabinofuranosyl-(1→6)-β-D-glucopyranoside, and ellagic acid exhibited the most potent proliferation activity of all.

## 1. Introduction

*Castanea crenata*, commonly referred to as Republic of Korean or Japanese chestnut, is an indigenous species of chestnut tree found in the Republic of Korea and Japan. The Republic of Korea ranks as the world’s second-highest producer of chestnuts, but 30% of them are processed manually as peeled chestnuts [1]. The process of peeling chestnuts generates two waste products: the burs and the shells. In Asia, the chestnut shell is used for its medicinal properties, including alleviating diarrhea and improving digestion [2]. Despite being a rich source of bioactive compounds, chestnut shells are considered solid waste and often burned as fuel in factories, leading to environmental problems caused by the release of poisonous substances such as CO, NOx, and polychlorodibenzodioxins [3]. Our previous studies have identified cycloartane-*type* triterpenoids and flavonoid glycosides in *C. crenata* burs, which have contributed to the discovery of natural antiviral sources [4]. Likewise, chestnut shells contain various health-beneficial compounds such as polyphenols and flavonoids, which have been shown to have antioxidant, anticancer, anti-inflammation, antibacterial, diabetes control, and weight loss effects [1,3,5].

Hair loss is a result of various factors, including aging, hormonal imbalances, nutrient deficiencies, and psychological stress. The hair follicle dermal papilla cells (DPCs) found at the base of the hair follicle are being employed in an in vitro screening model for hair growth, as they play a crucial role in hair follicular morphogenesis and postnatal hair growth cycles [6]. In our preliminary study, we discovered that (3*R*)-5′-methoxyvestiol and (6a*R*,11a*R*)-3,8-dihydroxy-9-methoxypterocarpan significantly impacted the proliferation of immortalized DPCs [7,8]. Additionally, chestnut shell extracts demonstrated minimal internal damage with excellent color intensity and a smooth hair surface morphology [5]. Thus, combining phytochemical exploration with biological activity investigation is crucial in discovering natural hair growth compounds from agricultural by-products. In this study, five previously undescribed compounds (**1**–**2**, **6**–**8**) along with seven known compounds were purified from *C. crenata* shells. Despite extensive investigation into the phenolic compounds in chestnut shells [2,9], no diterpenoid derivatives have been identified to date.

## 2. Results and Discussion

### 2.1. Phytochemical Isolation

**Figure 1:**
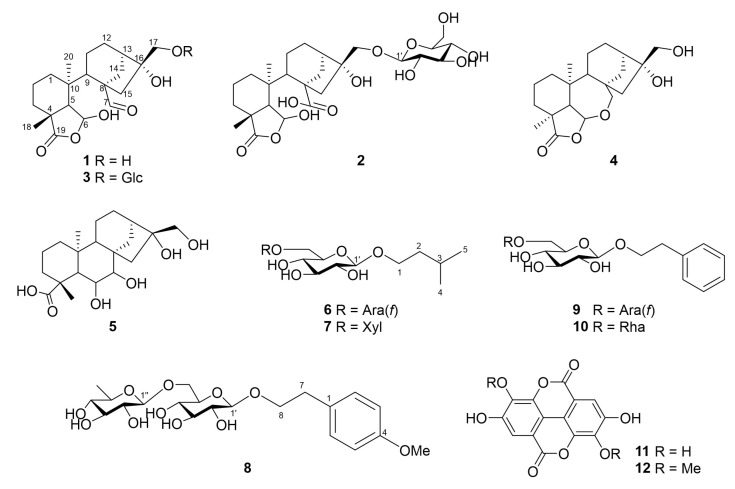
Chemical structures of compounds **1**–**12**.

In the methanol extract of *C. crenata* shells, five new compounds with seven known compounds were identified through column chromatography and isolation procedures (Figure 1). By comparing ^1^H and ^13^C NMR data to published data, the following compounds were characterized: prinsoside C (**3**) [10], mollissin (**4**) [11], 6β,7β,16α,17-tetrahydroxy-*ent*-kauranoic acid (**5**) [12], 2-phenylethyl 2-phenylethyl 6-*O*-α-L-arabinofuranosyl-β-D-glucopyranoside (**9**) [13], 2-phenylethyl β-rutinoside (**10**) [14], ellagic acid (**11**) [15], and 3,3′-di-*O*-methylellagic acid (**12**) [16] (Figure 1).

**Figure 2:**
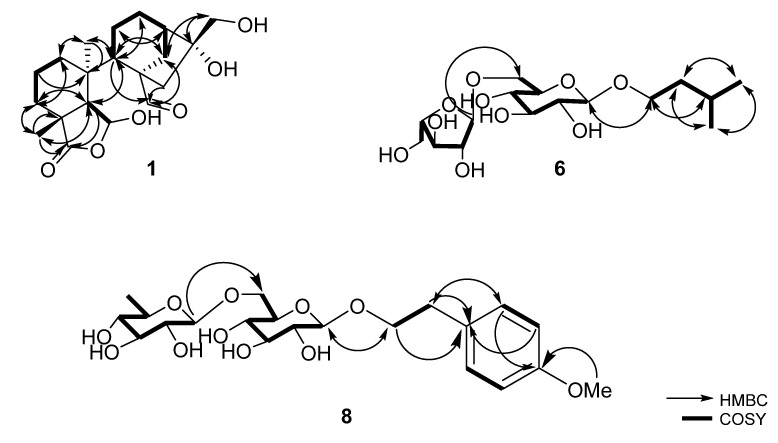
The key HMBC and COSY correlations of compounds **1**, **6**, and **8**.

**Figure 3:**
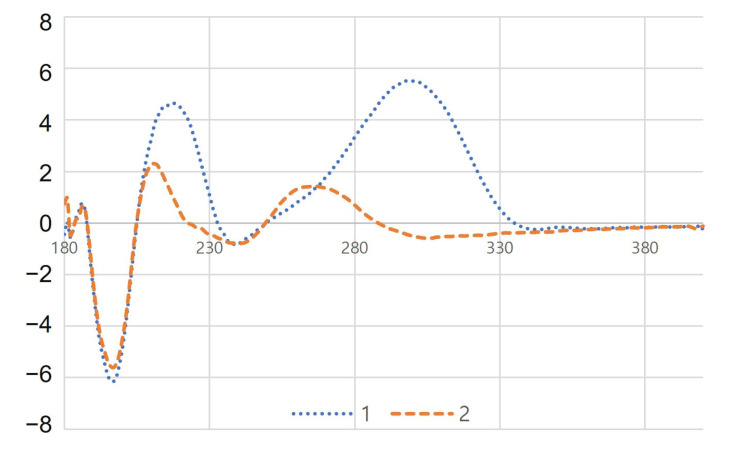
CD spectrum of compounds **1** and **2**.

Compound **1** was isolated from natural products for the first time and was previously found in the enzymatic hydrolysis form of prinsoside C [10]. It was purified as a white amorphous solid with a chemical formula of C_20_H_30_O_6_. The molecular ion [M–H]^–^ was found to be at *m*/*z* 365.1975 (calcd for C_20_H_29_O_6_, 365.1964, Figure S1). The ^1^H NMR spectra showed two methyls at *δ*_H_ 0.72 and 1.44 (both s), a hemiacetal at *δ*_H_ 5.67 (br s), and an aldehyde proton at *δ*_H_ 9.78 (s) (Figure S2). Two methyls (*δ*_C_ 22.6 and 30.6), eight methylenes (*δ*_C_ 19.0, 20.7, 26.4 32.1, 32.2 35.0, 47.7, and 66.1), three methines (*δ*_C_ 46.1, 48.9, and 55.8), one hemiacetal (*δ*_C_ 100.8), four quaternaries (*δ*_C_ 40.6, 42.4, 59.7, and 82.0), and two carbonyl carbons (*δ*_C_ 184.3 and 207.2) were detected (Table 1, Figure S3 and S4). With the exception of the sugar moiety, the NMR data of **1** indicated that it had structural similarity to prinsoside C, which has previously been isolated from *Prinsepia utilis* [10]. COSY correlations C-1/C-2/C-3, C-5/C-6, and C-11/C-12/C-13/C-14 indicated three coupling segments (Figure 2, Figure S5). The existence of a bicyclo [3.2.1]octane system in the HMBC spectrum was shown by the correlations between H-14 and C-9, C-12, C-15, C-16; H-11 and C-8, C-13; and H-13 and C-11. HMBC correlations between H-1 and C-20, C-3; H-6 and C-19; H-7 and C-8, C-9, C-14; Me-18 and C-3, C-4, C-5; and Me-20 and C-1, C-5, C-9, C-10 revealed an unusual B-ring *seco*-kaurane framework (Figure 2, Figure S6). Due to its biogenetic derivative, compound **1** was expected to have the same configurations at C-6 and C-16 as prinsoside C (**3**), which was also isolated from the present investigation. The CD spectra showed a negative Cotton effect at 239 nm (Δ*ε* –0.86, Figure 3), comparable to prinsoside C and *ent*-kaur-16-*en*-19-oic acid, which also showed the negative Cotton effect at 233 nm [10,17]. Based on these results, compound **1** was confirmed to be *ent*-6β,16α,17-trihydroxy-7,19-dioxo-6,19-epoxy-6,7-*seco*-kaurane and named prinsoside D.

The pseudo ion peak at *m/z* 543.2422 [M–H]^–^ revealed the molecular formula of **2** to be C_26_H_40_O_12_ (calcd for C_26_H_39_O_12_, 543.2442, Figure S7). The ^1^H NMR spectrum showed signals similar to those of prinsoside D (**1**): two methyl groups at *δ*_H_ 0.90 and 1.41 (both s) and a hemiacetal proton at *δ*_H_ 5.33 (br s) (Figure S8). The ^13^C NMR resonances were comparable to those of compound **1**, with the exception of a distinct signal set assignable to a sugar moiety carbon (*δ*_C_ 105.2, 78.1, 77.9, 75.3, 71.7, and 62.8) (Table 1, Figures S9 and S10). Except for the substitution of an aldehydic group for a carboxylic group at C-8, the NMR data of **2** were equivalent to those of prinsoside C [10]. The HMBC correlation between H-14, H-15, and C-7 verified the carboxylic group. Furthermore, the correlation between the anomeric proton at *δ*_H_ 4.29 and the methylene signal at *δ*_C_ 74.2 proved that the sugar moiety was located at C-17 (Figure 2, Figure S11). Compound **2** also exhibited the negative Cotton effect at 240 nm as with compound **1** (Figure 3). These results indicated that the structure of **2** could be determined as *ent*-6β,7,16α,17-tetrahydroxy-7,19-dioxo-6,19-epoxy-6,7-*seco*-kaurane 17-*O*-β-D-glucopyranoside (prinsoside E).

The ion peak at *m/z* 381.1939 [M–H]^–^ corresponded to the molecular formula of C_16_H_30_O_10_ (calcd for C_16_H_29_O_10_, 381.1761) of compound **6** (Figure S12). The existence of two sugar moieties and an aliphatic backbone was shown by the ^1^H and ^13^C NMR spectra. The ^1^H signals exhibited two methyls at *δ*_H_ 0.94 (6H, dd, *J* = 1.0, 6.6 Hz); two methylenes at *δ*_H_ 1.53 (2H), 3.59 (1H), and 3.93 (1H); and a methane at *δ*_H_ 1.77, suggesting the presence of isopentyl alcohol. Two anomeric protons at 4.27 (1H, d, *J* = 7.9 Hz) and 4.98 (1H, d, *J* = 1.5 Hz) were assigned to two sugar moieties with β- and α-linkages, respectively (Table 2, Figures S13 and S14). The 1→6 interglycoside linkage was confirmed by the glycosylation shift observed on C-6 of the first glucose unit and the HMBC correlation between H-1″ (*δ*_H_ 4.98) and C-6′ (*δ*_C_ 68.1) (Figure S15). The sugar moieties were identified as α-L-arabinofuranosyl and β-D-glucopyranoside by matching their NMR data to those of sugar moieties in 2-(4-hydroxyphenyl)ethyl-*O*-α-L-arabinofuranosyl-(1→6)-*O*-β-D-glucopyranoside [18]. Furthermore, HMBC correlations between H-1′ (*δ*_H_ 4.27) and C-1 (*δ*_C_ 69.4) confirmed the position of this sugar linkage at C-1 (Figure 2). Based on the data presented above, compound **6** was elucidated to be isopentyl-α-L-arabinofuranosyl-(1→6)-β-D-glucopyranoside.

The molecular formula of **7** was determined as C_16_H_30_O_10_ by the HR-ESI-MS ion at *m/z* 381.1899 [M−H]^−^ (calcd for C_16_H_29_O_10_, 381.1761, Figure S16). ^13^C NMR exhibited 11 signals assignable to primeverose [*O*-β-D-xylopyranosyl-(1→6)-*O*-β-D-glucopyranose] [19], two methyls, two methylenes, and one methine carbon signal. Its ^1^H and ^13^C NMR spectra revealed that it was similar to **6** (Figures S17 and S18), with the exception of the replacement of the arabinofuranosyl moiety with a xylopyranosyl moiety at C-6′. Consequently, structure **7** was determined as isopentyl β-D-primeverose.

The molecular formula of **8** was determined as C_21_H_32_O_11_ by the HR-ESI-MS ion at *m/z* 505.1928 [M+FA−H]^−^ (calcd for C_22_H_33_O_13_, 505.1921, Figure S19) Signals corresponding to two methylene groups at *δ*_H_ 2.87 (td, *J* = 4.7, 7.3 Hz, 2H) and *δ*_H_ 3.71 (dd, *J* = 7.7, 9.8 Hz, 1H) and 3.98 (dd, *J* = 7.7, 9.8 Hz, 1H) and aromatic protons at *δ*_H_ 6.82 (d, *J* = 8.3 Hz, 2H) and *δ*_H_ 7.16 (d, *J* = 8.3 Hz, 2H), as well as a methoxy proton at *δ*_H_ 3.45 (s, 3H), suggested a 2-(4-methoxyphenyl)ethanol backbone (Table 2, Figure S20). Two anomeric protons at *δ*_H_ 4.28 (1H, d, *J* = 7.7 Hz) and 4.74 (1H, d, *J* = 1.6 Hz) suggested β- and α-linkages of sugar moieties, respectively. The 13C-NMR spectrum revealed 20 carbon signals, including two quaternaries, fourteen methines, three methylenes, and one methyl carbon (Figure S21). The NMR data were comparable to those of tazettoside D [20], except for the substitution of glucopyranoside for rhamnopyranoside. The sugar moieties were identified to be a rutinoside (α-L-rhamnopyranosyl-(1→6)-β-D-glucopyranoside) by matching their NMR data with those of sugar moieties in isorhamnetin 3-*O*-rutinoside [21]. The HMBC correlations between H-1″ (*δ*_H_ 4.74) and C-6′ (*δ*_c_ 68.1) and between H-1′ (*δ*_H_ 4.28) and C-8 (*δ*_c_ 72.1) confirmed the presence of the sugar linkage to be 1→6 and the position of sugar moieties at C-8, respectively (Figure 2, Figure S22). Compound **8** was identified as 2-(4-methoxyphenyl)ethyl β-rutinoside as a result of the data presented above.

Among the known compounds, mollissin (**4**) was identified through the enzymatic hydrolysis of mollioside, a diterpene glycoside with a 6,7-secokauran-type carbon skeleton derived from the kernels of *C. mollissima* [11]. Ellagic acid (**11**) is abundant in various parts of *Castanea* species [9,22,23,24]. To the best of our knowledge, the remaining known compounds (**3**, **5**, **9**–**10**, **12**) are being reported for the first time in this species.

### 2.2. Dermal Papilla Cell Proliferation

Phytochemicals have been explored as potential hair growth stimulants due to their low toxicity, accessibility, affordability, and diverse modes of biochemical action [6,7,8]. Despite substantial studies on natural products for hair growth, the findings of compound-based research still require a more in-depth understanding, since the majority of these studies have used natural product extracts. In this investigation, the effects of all isolated compounds on dermal papilla cell proliferation were evaluated using a CCK-8 assay and the findings are summarized in Table 3.

Several compounds isolated from chestnut shells showed statistically significant effects on hair follicle proliferation, including prinsoside E (**2**), 6β,7β,16α,17-tetrahydroxy-*ent*-kauranoic acid, isopentyl-α-L-arabinofuranosyl-(1→6)-β-D-glucopyranoside, isopentyl β-D-primeverose, 2-(4-methoxyphenyl)ethyl β-rutinoside, 2-phenylethyl 6-*O*-α-L-arabinofuranosyl-β-D-glucopyranoside (**5**–**9**), and ellagic acid (**11**). Some of these compounds exhibited comparable proliferative activity to the positive control, minoxidil. The *ent*-kaurane-type diterpenoids, prinsoside E and 6β,7β,16α,17-tetrahydroxy-*ent*-kauranoic acid (**2** and **5**), exhibited the most promising activity, with proliferation of 133 ± 1.99 and 138 ± 2.54, respectively, which was better than that of the positive control, minoxidil (121 ± 5.85). These findings are supported by previous studies that found a substantial proliferative impact on human hair follicle dermal papilla cells from *ent*-kaurane-type diterpenoids isolated from Isodonis Herba [25].

Vanillic acid and hydroxytyrosol, simple phenolic acids found in wheat bran and olive oil, enhance DPCs’ proliferation. Vanillic acid stimulates anagen and reduces hair loss in DPCs, whereas hydroxytyrosol increases the release of hair growth factors during the anti-inflammatory process [26,27]. Research into the effects of phenolic glycosides on hair growth has not been extensively conducted. Nevertheless, all the phenolic glycosides isolated from the chestnut shells, except 2-phenylethyl β-rutinoside (**10**), in the present study displayed significant activity, with proliferation ranging from 117 to 138.

In accordance with the results from the present investigation, previous research has demonstrated that ellagic acid (**11**) has a significant effect in stimulating hair growth by extending the follicular size and prolonging the growing phase [28]. Additionally, ellagic acid protected irradiated hair follicle dermal papilla cells from UVA-induced damage by exhibiting an ROS scavenging capacity and modulating antioxidant gene expression [29]. These findings support the potential use of chestnut by-products as a source of high-value compounds and the possibility that diterpenoids and phenolic glycosides derived from chestnut shells might be promising candidates for hair-growth-enhancing agents. Further research is being undertaken to understand the mechanism behind their proliferation effects.

## 3. Materials and Methods

### 3.1. Plant Material

*Castanea crenata* shells from Gongju-si, Chungcheongnam-do, Republic of Korea, were obtained from the Kyung-dong herbal market in Seoul, Republic of Korea, in 2019. The Okkwang cultivar, widely cultivated in the Republic of Korea, was used for this study [2]. A voucher specimen (CC201901) was deposited in the Herbarium of the College of Pharmacy, Yonsei Institute of Pharmaceutical Sciences, Yonsei University, Incheon, Republic of Korea.

### 3.2. Extraction and Isolation

*C. crenata* dried shells (10.0 kg) were extracted with MeOH (5 L × 3 times) and sonicated for 4 h at 30 °C, yielding a 200.0 g extract. The extract was then partitioned with CHCl_3_ and EtOAc, resulting in CHCl_3_ (CCS1), EtOAc (CCS2), and H_2_O (CCS3) fractions. The CHCl_3_ fraction (CCS1) was further fractionated using a silica gel CC with a gradient of *n*-hexane:acetone (20:1→1:1, *v*/*v*) to give five sub-fractions (CCS1A–CCS1E). The CCS1C fraction was again applied to a silica gel column with CHCl_3_:MeOH (4:1, *v*/*v*) to produce five additional fractions (CCS1C1–CCS1C5). The CCS1C2 fraction yielded **1** (26.4 mg) and **12** (12.8 mg) using preparative HPLC with a J’sphere ODS H-80 column (250 mm × 20 mm), eluted with 20% MeCN in H_2_O at a flow rate of 3 mL/min. Under the same HPLC conditions, the CCS1C3 fraction purified **6** (16.0 mg), **7** (10.6 mg), **8** (13.0 mg), **9** (17.2 mg), and **10** (13.0 mg), while the CCS1C4 fraction purified **2** (9.1 mg), **3** (9.4 mg), **5** (2.8 mg), and **11** (2.7 mg). The EtOAc fraction (CCS2) was fractionated on a silica gel column with a gradient of CHCl_3_:MeOH (20:1→1:1, *v*/*v*), yielding five sub-fractions (CCS2A–CCS2E). The CCS2C fraction was further processed on a YMC RP-18 column with MeOH:H_2_O (2:1, *v*/*v*) to produce two smaller fractions (CCS2C1 and CCS2C2). The CCS2C1 fraction yielded **4** (3.1 mg) using the HPLC procedure described above, with 15% MeCN in water as the solvent.

#### 3.2.1. Prinsoside D (**1**)

White amorphous solid; CD (*c* = 2 × 10^−4^, MeOH) Δ*ε* (nm) –0.86 (239); C_20_H_30_O_6_, HR-ESI-MS *m*/*z*: 365.1975 [M–H]^–^ (calcd for C_20_H_29_O_6_, 365.1964); for ^1^H (CD_3_OD, 600 MHz) and ^13^C NMR (CD_3_OD, 150 MHz); spectroscopic data, see Table 1.

#### 3.2.2. Prinsoside E (**2**)

White amorphous solid; CD (*c* = 2 × 10^−4^, MeOH) Δ*ε* (nm) –0.80 (240); C_26_H_40_O_12_, HR-ESI-MS *m*/*z*: 543.2422 [M–H]^–^ (calcd for C_26_H_39_O_12_, 543.2442); for ^1^H (CD_3_OD, 600 MHz) and ^13^C NMR (CD_3_OD, 150 MHz); spectroscopic data, see Table 1.

#### 3.2.3. Isopentyl-α-L-arabinofuranosyl-(1→6)-β-D-glucopyranoside (**6**)

White amorphous powder; C_16_H_30_O_10_, HR-ESI-MS *m*/*z*: 381.1939 [M–H]^−^ (calcd for C_16_H_29_O_10_, 381.1761); for ^1^H (CD_3_OD, 600 MHz) and ^13^C NMR (CD_3_OD, 150 MHz); spectroscopic data, see Table 2.

#### 3.2.4. Isopentyl β-D-primeverose (**7**)

White amorphous powder; C_16_H_30_O_10_, HR-ESI-MS *m/z*: 381.1899 [M−H]^−^ (calcd for C_16_H_29_O_10_, 381.1761); for ^1^H (CD_3_OD, 600 MHz) and ^13^C NMR (CD_3_OD, 150 MHz); spectroscopic data, see Table 2.

#### 3.2.5. 2-(4-Methoxyphenyl)ethyl β-Rutinoside (**8**)

White amorphous powder; C_21_H_32_O_11_, HR-ESI-MS *m/z*: 505.1928 [M+FA−H]^−^ (calcd for C_22_H_33_O_13_, 505.1921); for ^1^H (CD_3_OD, 600 MHz) and ^13^C NMR (CD_3_OD, 150 MHz); spectroscopic data, see Table 2.

### 3.3. Cell Culture and Cell Proliferation Assay

Immortalized dermal papilla cells (iDPCs) were obtained from Dr. Sung’s Lab at Kyungpook National University and cultured in DMEM containing 10% FBS (Gibco, Rockville, MD, USA) and 1% penicillin/streptomycin (Gibco, CA, USA) at 37 °C in a humidified atmosphere of 95% air/5% CO_2_. A total of 1000 iDPCs cells per well were plated in 96-well microplates and treated with 30 μM of isolated compounds in DMSO as well as minoxidil, the positive control, for 24 h. The cell proliferation was measured using the CCK-8 solution (Dojindo Molecular Technologies, Inc., Rockville, MD, USA). The absorbance was measured at 450 nm using a microplate reader (Tecan, AG, Switzerland). The percentage of cell proliferation was calculated by setting the control group without compound (blank) as 100%.

### 3.4. Statistical Analysis

The statistical software package GraphPad Prism (ver. 5, GraphPad, San Diego, CA, USA) was used to examine the data. All data are presented as means ± standard error of the mean (SEM). The statistical significance of the differences between the compounds and the control group (blank) was determined using a paired *t*-test. *p*-values were considered statistically significant when <0.05 (*), <0.01 (**), and <0.001 (***).

## 4. Conclusions

The phytochemical investigation of the shells of *C. crenata* resulted in the identification of five new compounds (**1**–**2**, **6**–**8**) and seven previously described compounds (**3**–**5**, **9**–**12**). To the best of our knowledge, this is the first work on diterpene separation from *C. crenata* shells. Twelve compounds obtained from *C. crenata* shells were investigated for iDPC proliferation. In particular, prinsoside E (2), 6β,7β,16α,17-tetrahydroxy-*ent*-kauranoic acid, isopentyl-α-L-arabinofuranosyl-(1→6)-β-D-glucopyranoside (**5**–**6**), 2-(4-methoxyphenyl)ethyl β-rutinoside (**8**), and ellagic acid (**11**) demonstrated significant proliferative effects on iDPC. These effects were comparable to those of the positive control, minoxidil. These findings suggest that chestnut shells might be utilized in the treatment of hair loss and further studies on the target mechanism of these constituents are currently in progress.

## Figures and Tables

**Table 1:** NMR spectroscopic data for compounds **1**–**2**.

Pos	1		2	
	*δ*_C_^a,b^	*δ*_H_^a,c^ (*J* in Hz)	*δ*_C_^a,b^	*δ*_H_^a,c^ (*J* in Hz)
1	35.0	1.59 (m), 1.75 (m)	35.1	1.55 (m)
2	19.0	1.44 (m)	19.1	1.44 (m)
3	32.1	1.32 (m), 2.07 (m)	32.2	1.27 (m), 2.07 (m)
4	42.4	-	42.2	-
5	55.8	1.94 (m)	56.2	1.86 (m)
6	100.8	5.67 (s)	100.9	5.33 (s)
7	207.2	9.78 (s)	182.3	-
8	59.6	-	54.1	-
9	48.9	2.09 (m)	48.9	2.07 (m)
10	40.5	-	39.7	-
11	20.7	1.83 (m)	20.8	1.74 (m)
12	26.4	1.70 (m)	25.6	1.57 (m), 1.68 (m)
13	46.1	2.25 (m)	46.3	2.21 (m)
14	32.2	1.70 (m), 2.42 (dd, 4.5, 12.0)	33.2	1.66 (m), 2.53 (m)
15	47.7	1.60 (m), 1.83 (m)	51.9	1.75 (m), 2.03 (m)
16	82.0	-	81.4	-
17	66.1	3.67 (d, 11.4), 3.75 (d, 11.4)	74.2	3.55 (dd, 1.7, 10.3), 4.21 (dt, 1.7, 10.3)
18	30.6	1.44 (s)	30.9	1.41 (s)
19	184.3	-	184.6	-
20	22.6	0.72 (s)	18.5	0.90 (s)
1′			105.2	4.29 (dt, 1.6, 7.8)
2′			78.1	3.27 *
3′			75.3	3.22 (ddd, 1.6, 7.6, 9.3)
4′			71.7	3.27 *
5′			77.9	3.36 *
6′			62.8	3.67 *, 3.88 (dd, 1.3, 11.7)

^a^ Measured in CD_3_OD, ^b^ 150 MHz, ^c^ 600 MHz, * overlapped, assignments were done by HSQC, HMBC, COSY, and ROESY experiments.

**Table 2:** NMR spectroscopic data for compounds **6**–**8**.

Pos	6		7		8	
	*δ*_C_^a,b^	*δ*_H_^a,c^ (*J* in Hz)	*δ*_C_^a,b^	*δ*_H_^a,c^ (*J* in Hz)	*δ*_C_^a,b^	*δ*_H_^a,c^ (*J* in Hz)
1	69.4	3.59 (m), 3.93 (m)	69.4	3.56 (dt, 6.9, 9.6), 3.92 (dt, 6.9, 9.6)	131.9	-
2	39.6	1.53 (q, 6.9)	39.6	1.51 (dt, 6.9, 8.2),	130.9	7.19 (d, 8.3)
3	26.0	1.77 (dt, 6.7, 13.4)	26.0	1.75 (dt, 6.8, 13.4)	114.8	6.82 (d, 8.3)
4	23.0	0.94 (dd, 1.0, 6.6)	23.0	0.92 (d, 6.8)	159.6	-
5	23.1	0.94 (dd, 1.0, 6.6)	23.1	0.92 (d, 6.8)	114.8	6.82 (d, 8.3)
6					130.9	7.19 (d, 8.3)
7					36.4	2.87 (td, 4.7, 7.3)
8					72.1	3.71 (dd, 7.7, 9.8), 3.98 (dd, 7.7, 9.8)
OMe					55.6	3.45 (s)
1′	104.4	4.27 (d, 7.9)	104.4	4.24 (d, 7.9)	104.5	4.28 (d, 7.7)
2′	75.1	3.18 (dd, 7.9, 9.3)	75.1	3.17 (m)	75.1	3.16 (m)
3′	78.0	3.36 *	78.0	3.34 (m)	78.0	3.33 *
4′	72.0	3.30 *	72.0	3.32 (m)	71.6	3.27 *
5′	76.6	3.45 *	76.6	3.43 (m)	77.1	3.38 *
6′	68.1	3.61 *, 3.96 (dd, 2.4, 11.2)	69.7	3.74 (dd, 6.0, 11.5), 4.08 (dd, 2.2, 11.5)	68.1	3.61 (dd, 6.2, 11.3), 3.96 *
1″	109.9	4.98 (d, 1.5)	105.5	4.32 (d, 7.5)	102.4	4.74 (d, 1.6)
2″	83.2	4.01 (dd, 1.5, 3.3)	74.8	3.21 (dd, 7.5, 9.0)	72.2	3.82 *
3″	78.9	3.84 (dd, 3.2, 11.9)	77.6	3.31 (m)	72.4	3.65 *
4″	85.9	3.99 (m)	71.1	3.48 (m)	74.0	3.36 *
5″	63.1	3.66 *, 3.76 (dd, 3.2, 11.9)	66.9	3.19 (dd, 3.2, 11.9), 3.87 (dd, 3.2, 11.9)	69.7	3.66 *
6″					18.1	1.25 (d, 6.2)

^a^ Measured in CD_3_OD, ^b^ 150 MHz, ^c^ 600 MHz, * overlapped, assignments were done by HSQC, HMBC, COSY, and ROESY experiments.

**Table 3:** Dermal papilla cell proliferation effect of isolated compounds.

Isolated Compounds	Proliferation (%)
Prinsoside D (**1**)	90 ± 3.15
Prinsoside E (**2**)	133 ± 1.99 ^b^
Prinsoside C (**3**)	105 ± 2.53
Mollissin (**4**)	87 ± 2.40
6β,7β,16α,17-Tetrahydroxy-*ent*-kauranoic acid (**5**)	138 ± 2.54 ^c^
Isopentyl-α-L-arabinofuranosyl-(1→6)-β-D-glucopyranoside (**6**)	138 ± 4.41 ^c^
Isopentyl β-D-primeverose (**7**)	117 ± 5.08 ^a^
2-(4-Methoxyphenyl)ethyl β-rutinoside (**8**)	123 ± 5.27 ^a^
2-Phenylethyl 6-O-α-L-arabinofuranosyl-β-D-glucopyranoside (**9**)	118 ± 2.10 ^a^
2-Phenylethyl β-rutinoside (**10**)	99 ± 2.68
Ellagic acid (**11**)	137 ± 2.29 ^c^
3,3′-Di-O-methylellagic acid (**12**)	60 ± 1.50
Minoxidil *	121 ± 5.85 ^a^

The absorbance at 450 nm was measured using a microplate reader. Mean ± S.E.M. * Positive control. The percentage of proliferation was calculated by setting the control group without compound (blank) as 100%. ^a^ *p* < 0.05; ^b^ *p* < 0.01; ^c^ *p* < 0.001 indicate statistically significant differences compared to the control group.

## Data Availability

The data presented in this study are available on request from the corresponding author.

## Supplementary Materials

The following supporting information can be downloaded at: https://www.mdpi.com/article/10.3390/plants12051018/s1, Figure S1 HR-ESI-MS of compound **1**; Figure S2 ^1^H-NMR spectrum of compound **1**; Figure S3 ^13^C-NMR spectrum of compound **1**; Figure S4 HSQC spectrum of compound **1**; Figure S5 COSY of compound **1**; Figure S6 HMBC of compound **1**; Figure S7 HR-ESI-MS of compound **2**; Figure S8 ^1^H-NMR spectrum of compound **2**; Figure S9 ^13^C-NMR spectrum of compound **2**; Figure S10 HSQC spectrum of compound **2**; Figure S11 HMBC spectrum of compound **2**; Figure S12 HR-ESI-MS of compound **6**; Figure S13 ^1^H-NMR spectrum of compound **6**; Figure S14 ^13^C-NMR spectrum of compound **6**; Figure S15 HMBC spectrum of compound **6**; Figure S16 HR-ESI-MS of compound **7**; Figure S17 ^1^H-NMR spectrum of compound **7**; Figure S18 ^13^C-NMR spectrum of compound **7**; Figure S19 HR-ESI-MS of compound **8;** Figure S20 ^1^H-NMR spectrum of compound **8**; Figure S21 ^13^C-NMR spectrum of compound **8**; Figure S22 HMBC spectrum of compound **8**.
