# Supplementary material for: Dermal Papilla Cell Proliferation of Phytochemicals Isolated from Chestnut Shells (Castanea crenata)
Source: Plants (Basel). 2023 Feb 23;12(5):1018. doi: 10.3390/plants12051018 (PMC10005320; doi:10.3390/plants12051018)
Supplement: Supplementary file 1 [file plants-12-01018-s001.zip › plants-2202548-supplementary.pdf]

Dermal Papilla Cell Proliferation of Phytochemicals Isolated from Chestnut Shells (*Castanea crenata*)

SeonJu Park [1], Nahyun Choi [2], Le Nu Huyen Trang [2], Mira Oh [3,4], Youngse Oh [3], Jong-Hyuk Sung [2,3], and Seung Hyun Kim [3,*]

[1] Chuncheon Center, Korea Basic Science Institute (KBSI), Chuncheon 24341, Republic of Korea

[2] Epibiotech Co., Ltd., Incheon 21984, Republic of Korea

[3] College of Pharmacy, Yonsei Institute of Pharmaceutical Sciences, Yonsei University, Incheon 21983, Republic of Korea

[4] Research Group of Traditional Food, Korea Food Research Institute, Wanju-gun 55365, Korea

[*] Correspondence: kimsh11@yonsei.ac.kr

List of Contents

no.	Content	Page
1	**Figure S1** HR-ESI-MS of compound **1**	S3
2	**Figure S2** ^{1}H-NMR spectrum of compound **1**	S4
3	**Figure S3** ^{13}C-NMR spectrum spectrum of compound **1**	S5
4	**Figure S4** HSQC spectrum of compound **1**	S6
5	**Figure S5** COSY of compound **1**	S7
6	**Figure S6** HMBC of compound **1**	S8
7	**Figure S7** HR-ESI-MS of compound **2**	S9
8	**Figure S8** ^{1}H-NMR spectrum of compound **2**	S10
9	**Figure S9** ^{13}C-NMR spectrum of compound **2**	S11
10	**Figure S10** HSQC spectrum of compound **2**	S12
11	**Figure S11** HMBC spectrum of compound **2**	S13
12	**Figure S12** HR-ESI-MS of compound **6**	S14
13	**Figure S13** ^{1}H-NMR spectrum of compound **6**	S15
14	**Figure S14** ^{13}C-NMR spectrum of compound **6**	S16
15	**Figure S15** HMBC spectrum of compound **6**	S17
16	**Figure S16** HR-ESI-MS of compound **7**	S18
17	**Figure S17** ^{1}H-NMR spectrum of compound **7**	S19
18	**Figure S18** ^{13}C-NMR spectrum of compound **7**	S20
19	**Figure S19** HR-ESI-MS of compound **8**	S21
20	**Figure S20** ^{1}H-NMR spectrum of compound **8**	S22
21	**Figure S21** ^{13}C-NMR spectrum of compound **8**	S23
22	**Figure S22** HMBC spectrum of compound **8**	S24

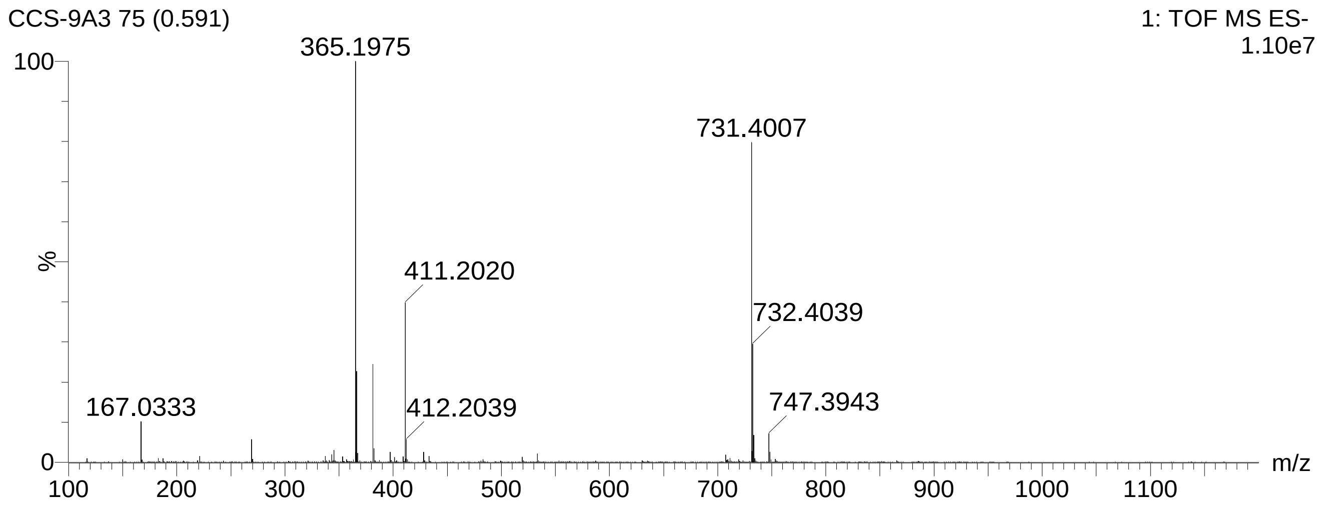

Figure S1 HR-ESI-MS of compound **1**

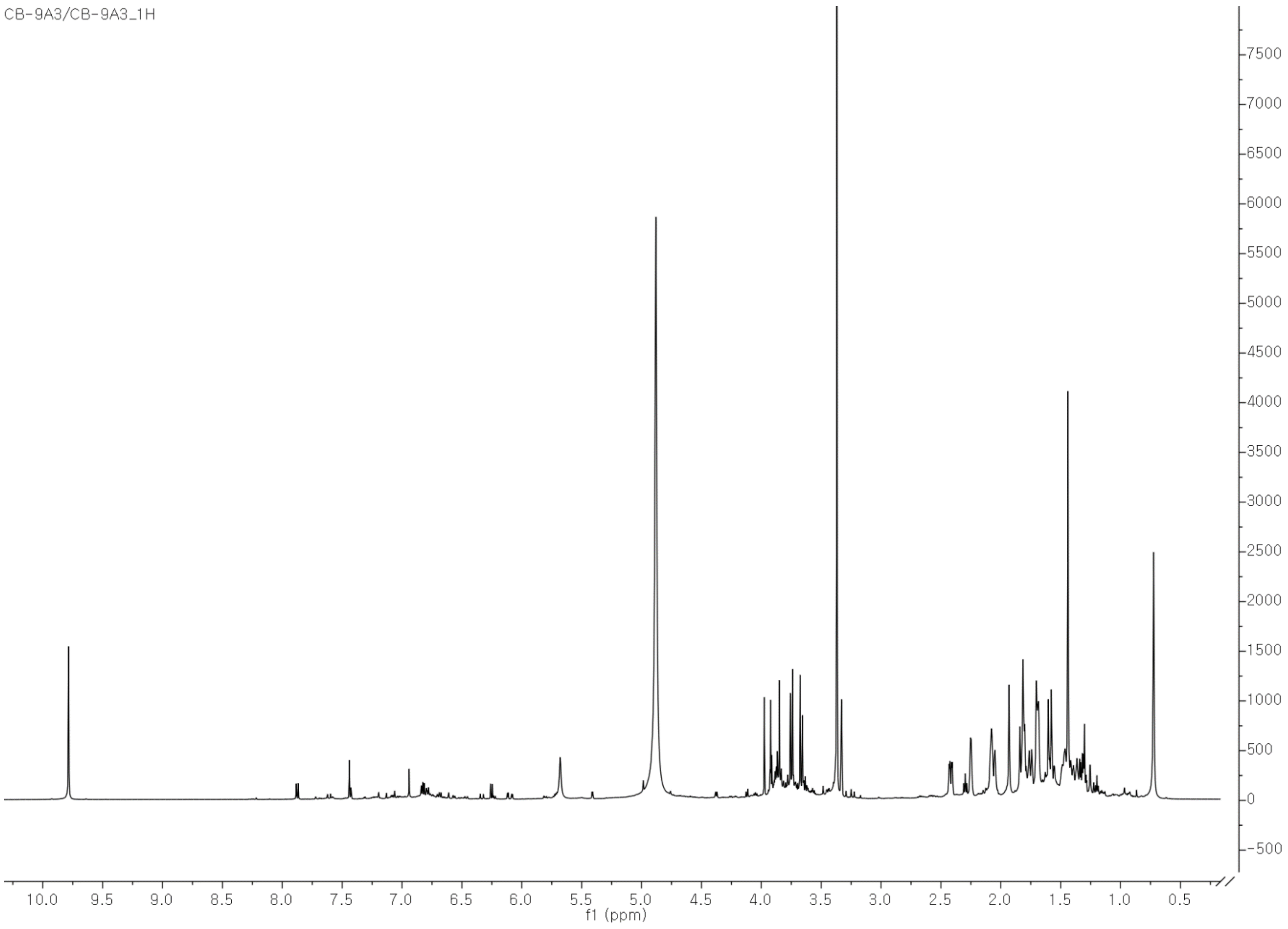

Figure S2 ^{1}H-NMR spectrum of compound **1** (600 MHz, methanol-d_4)

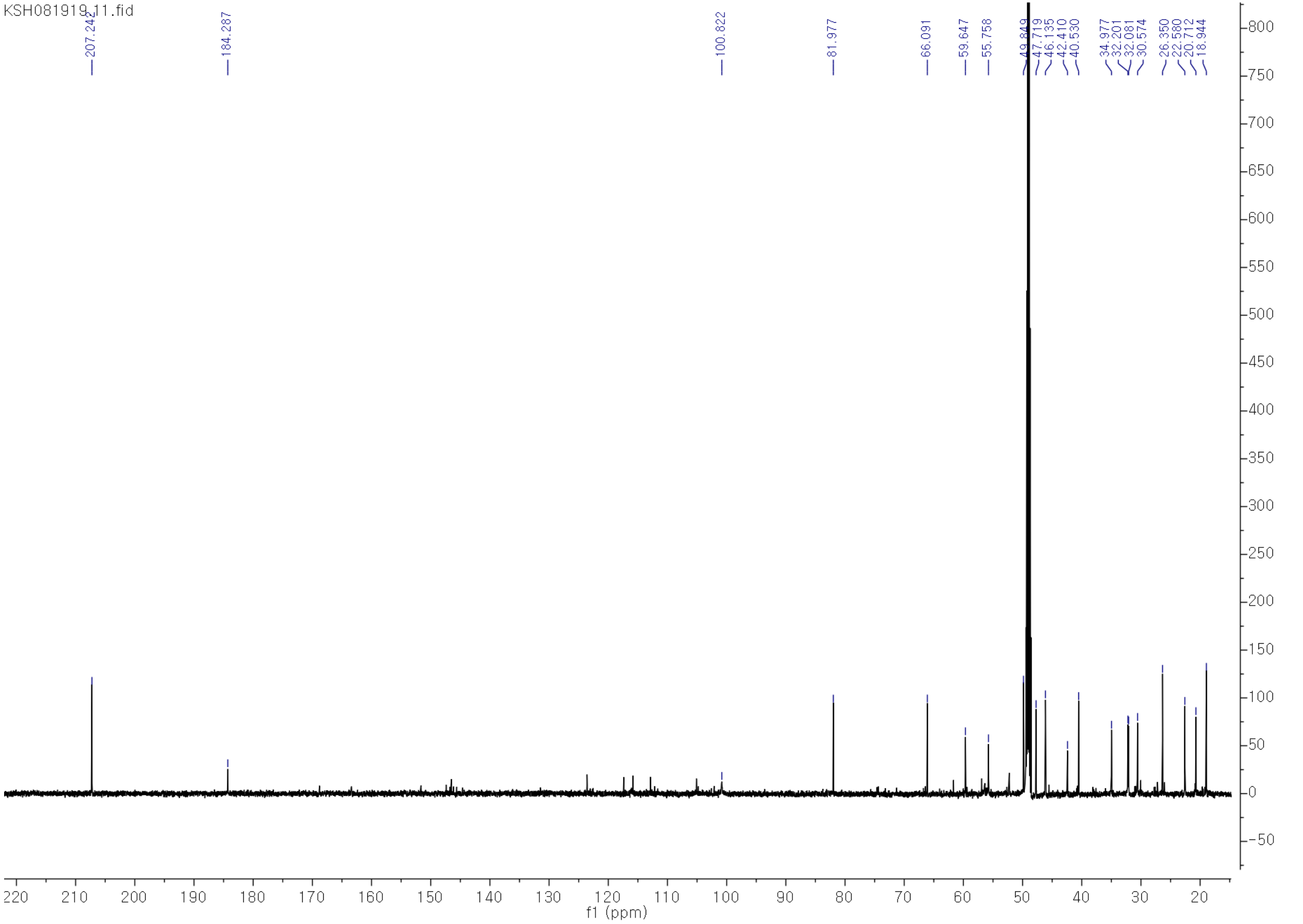

Figure S3 ^{13}C-NMR spectrum of compound **1** (150 MHz, methanol-d_4)

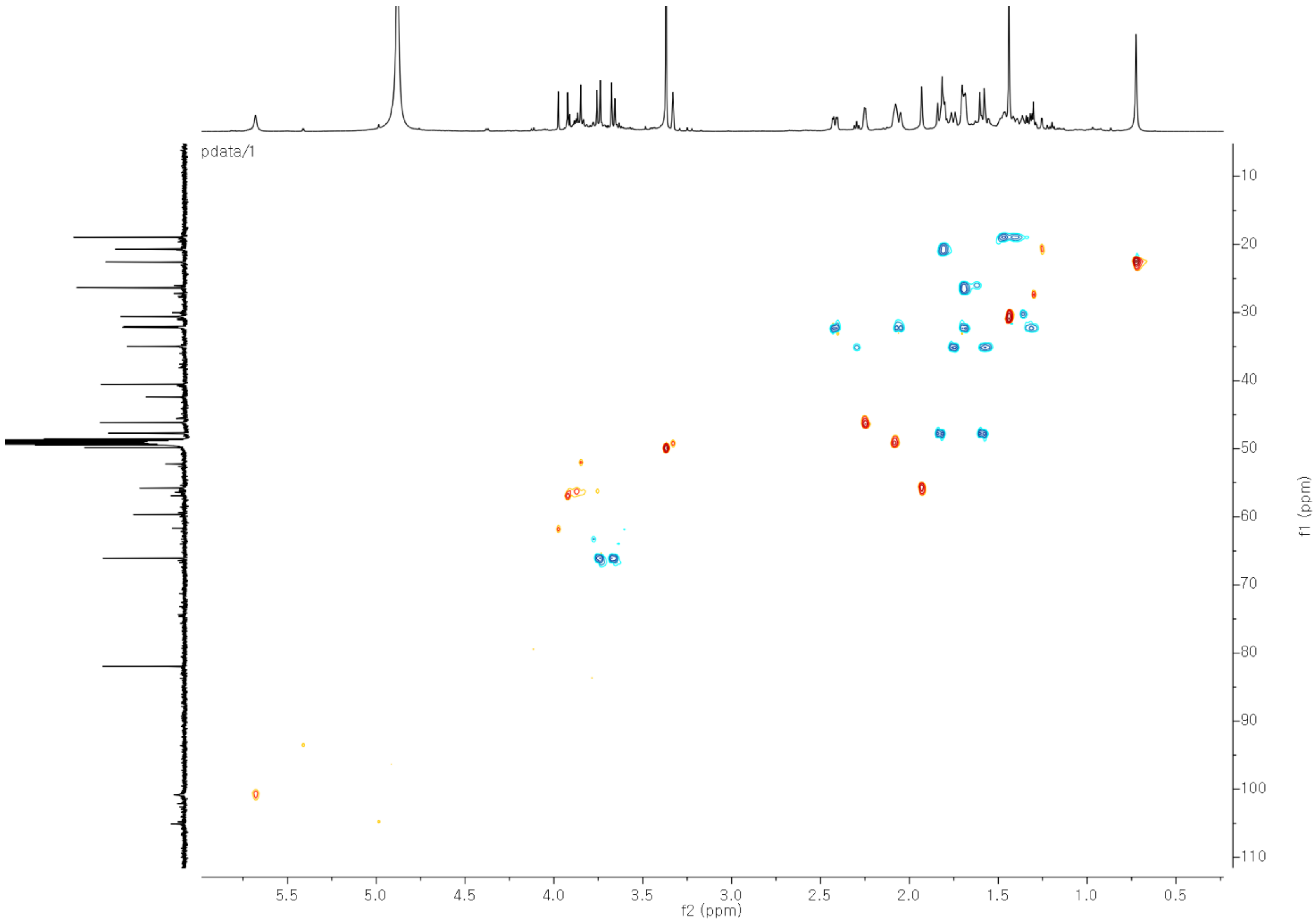

Figure S4 HSQC spectrum of compound **1** (methanol-d_4)

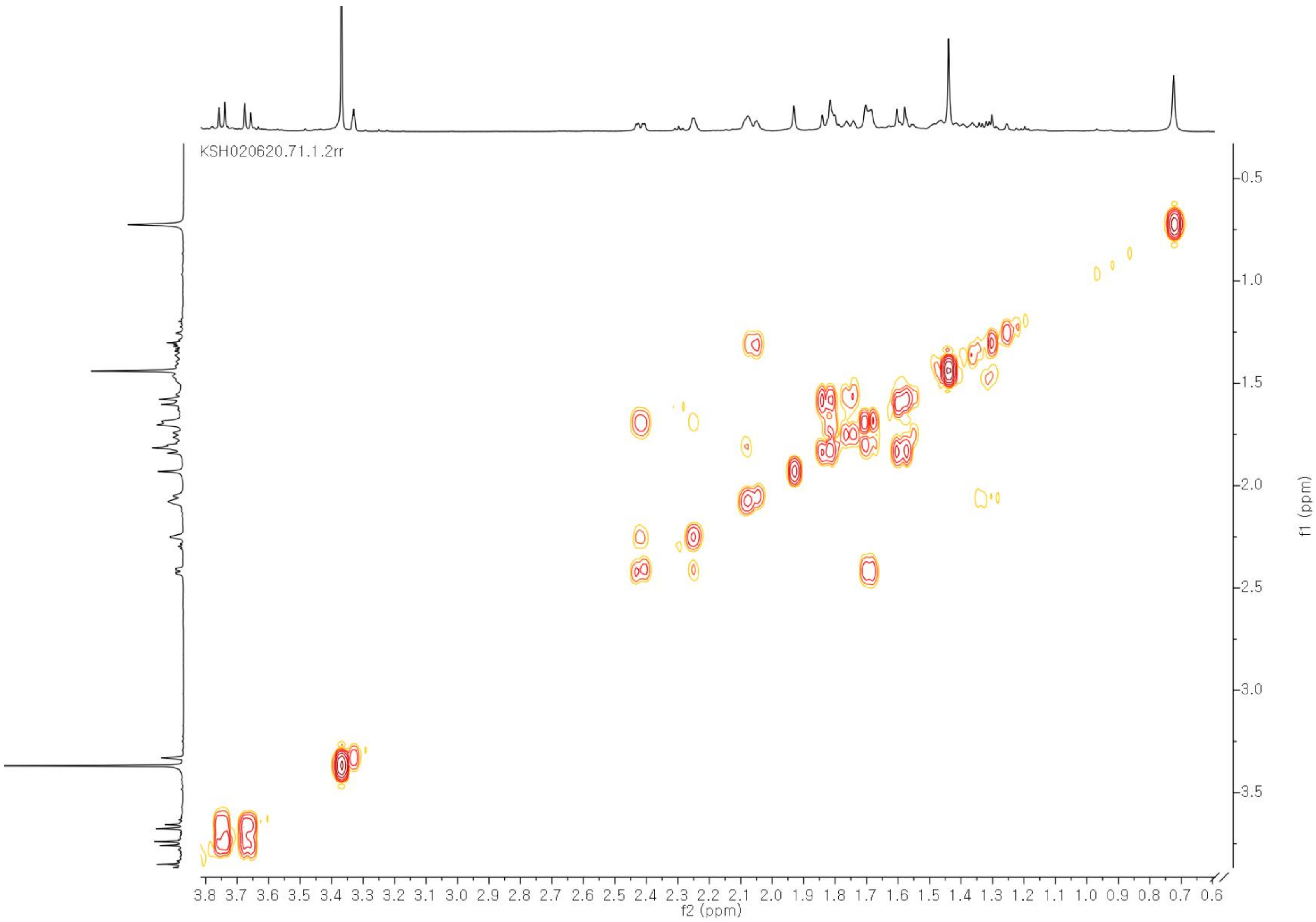

Figure S5 COSY of compound **1** (methanol-d_4)

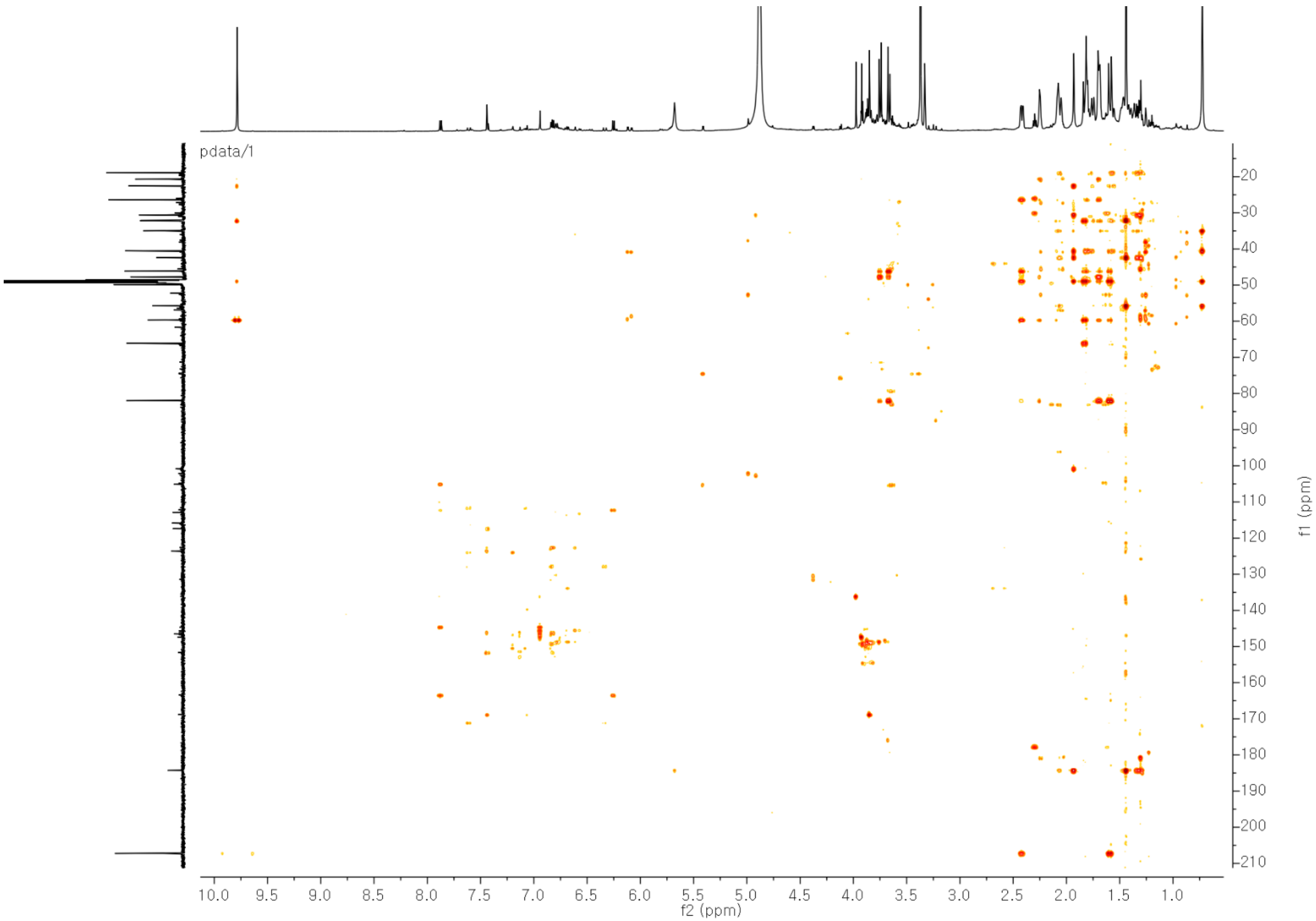

Figure S6 HMBC spectrum of compound **1** (methanol-d_4)

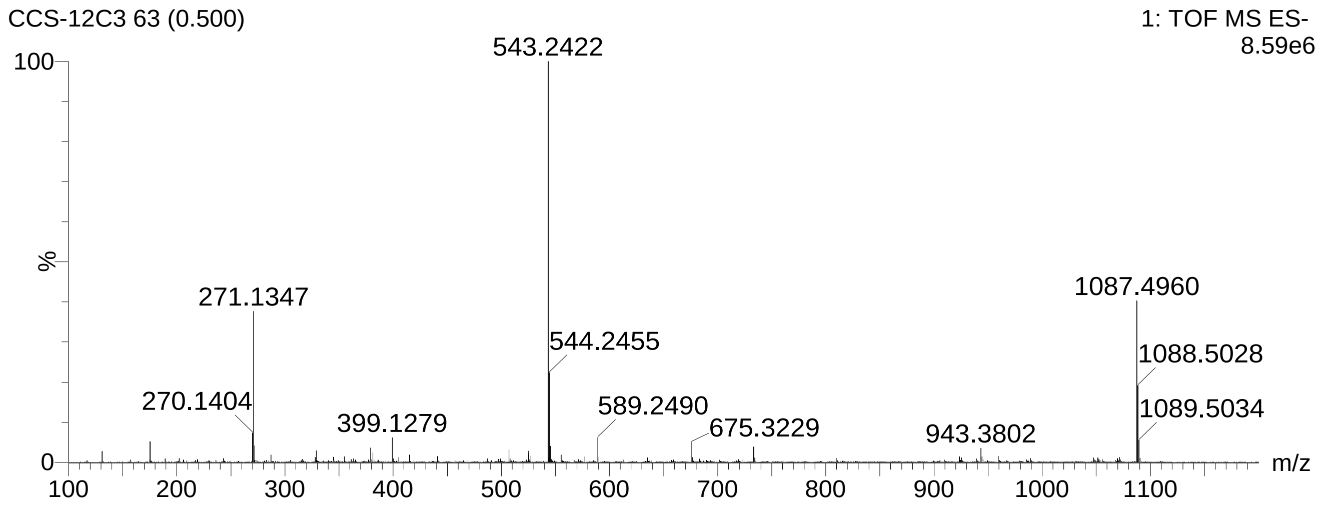

Figure S7 HR-ESI-MS of compound **2**

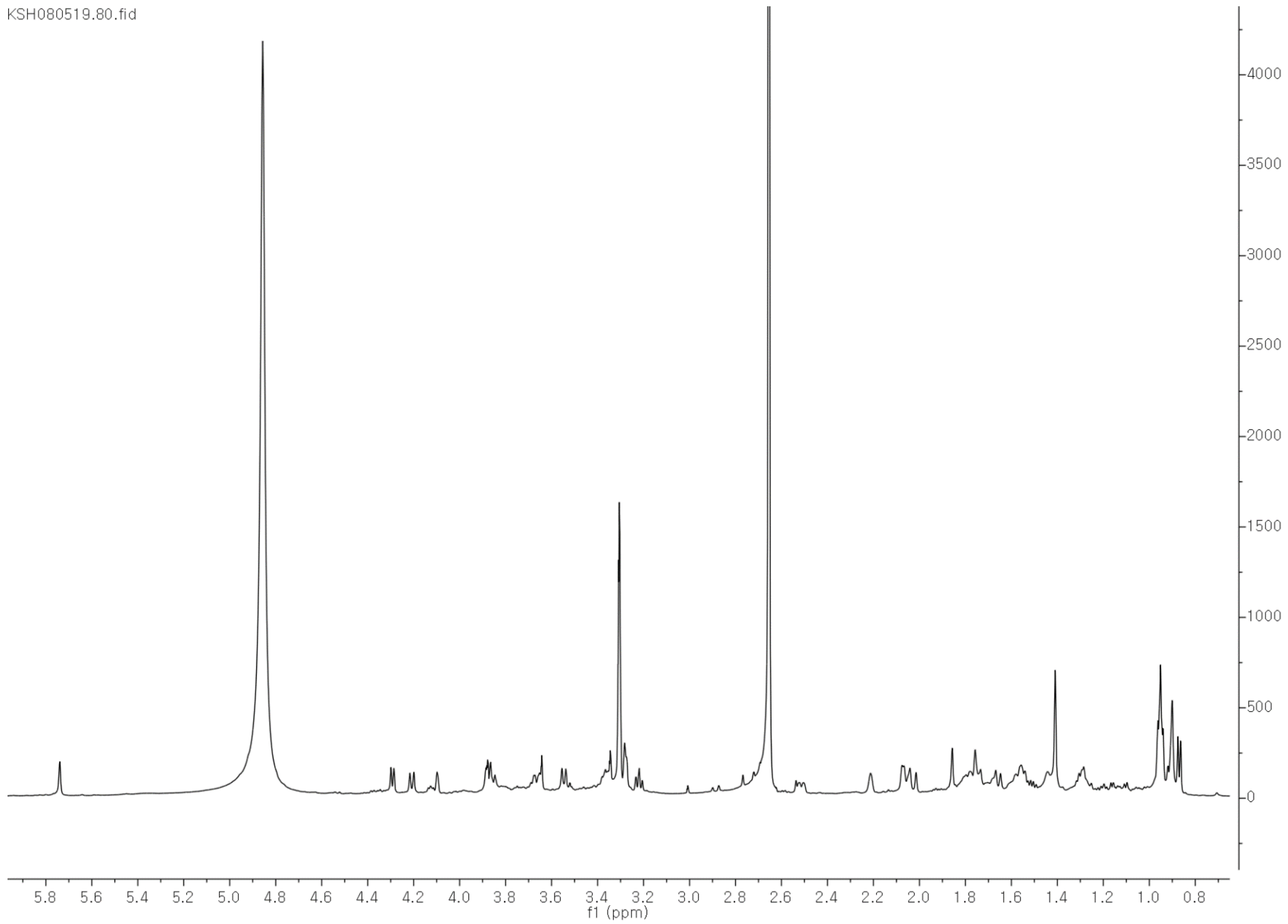

Figure S8 ^{1}H-NMR spectrum of compound **2** (600 MHz, methanol-d_4)

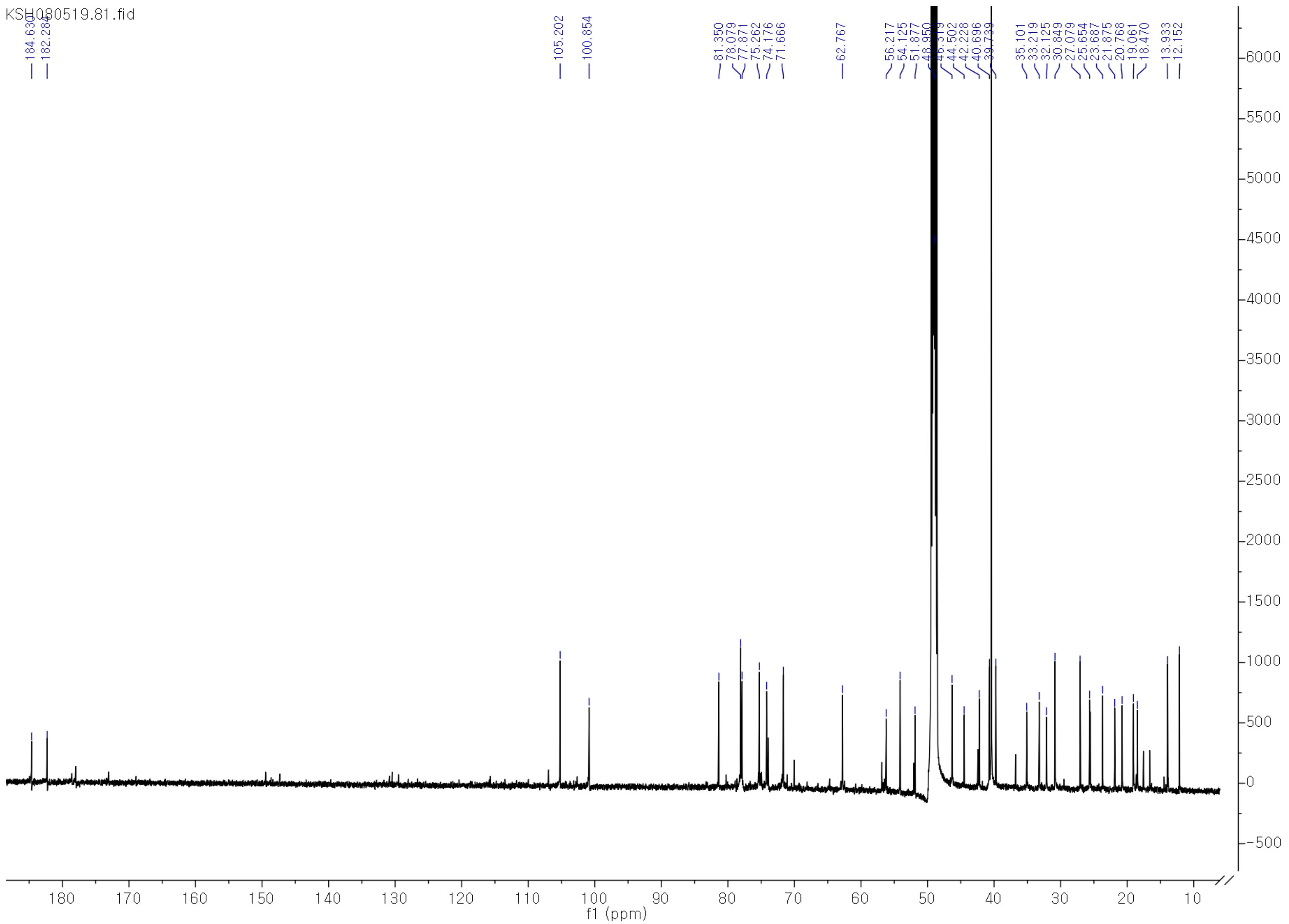

Figure S9 ^{13}C-NMR spectrum of compound **2** (150 MHz, methanol-d_4)

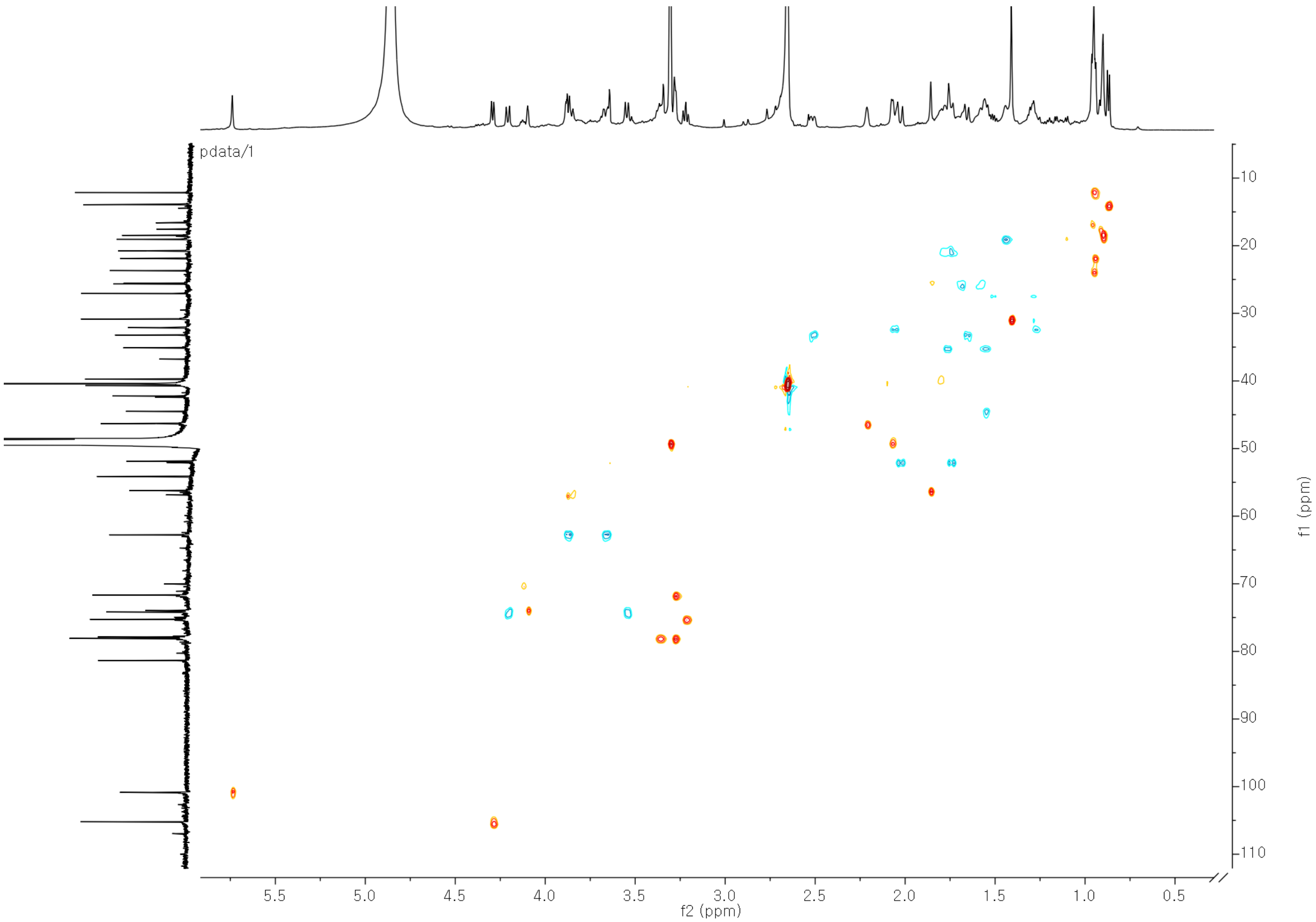

Figure S10 HSQC spectrum of compound **2** (methanol-d_4)

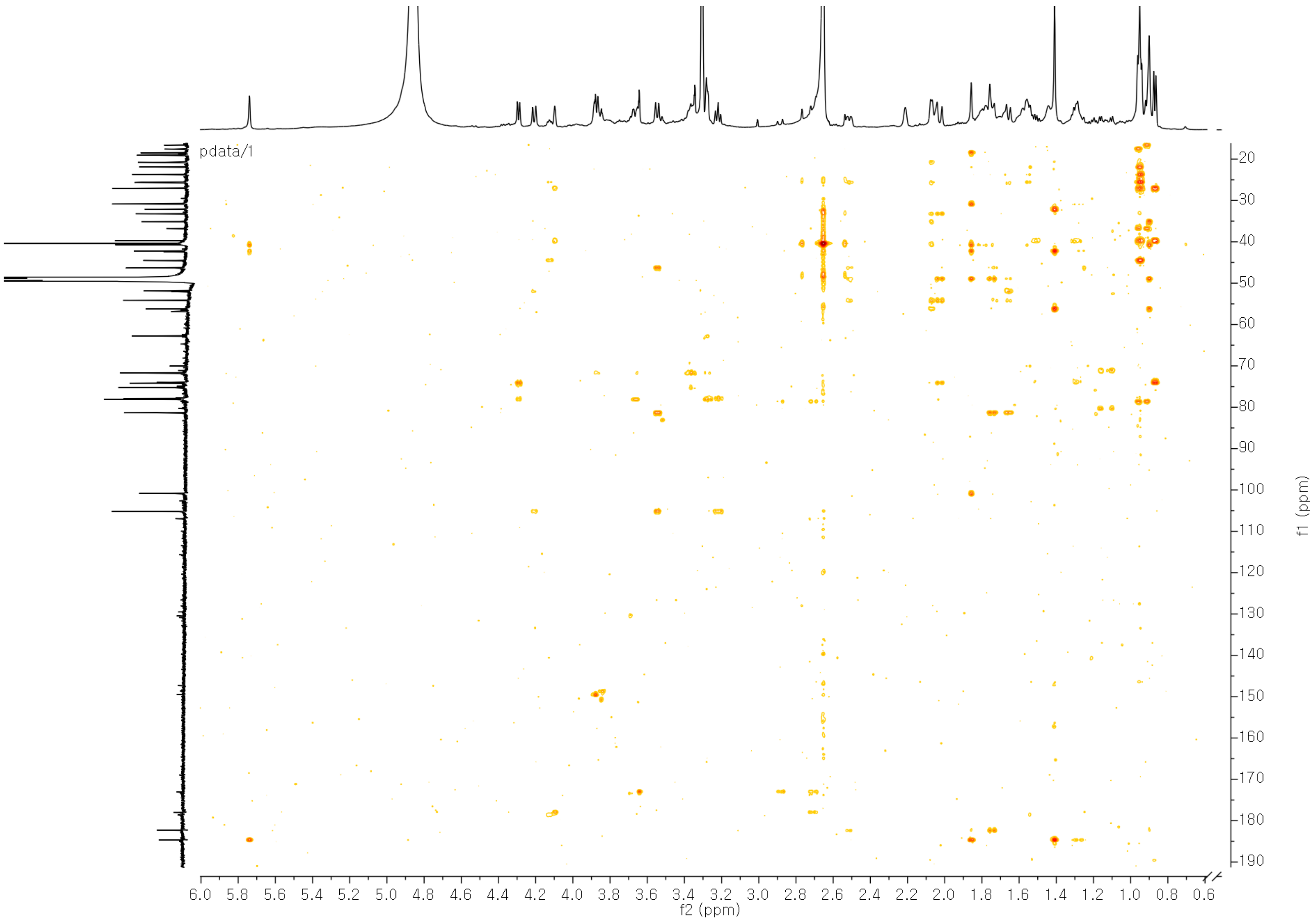

Figure S11 HMBC spectrum of compound **2** (methanol-d_4)

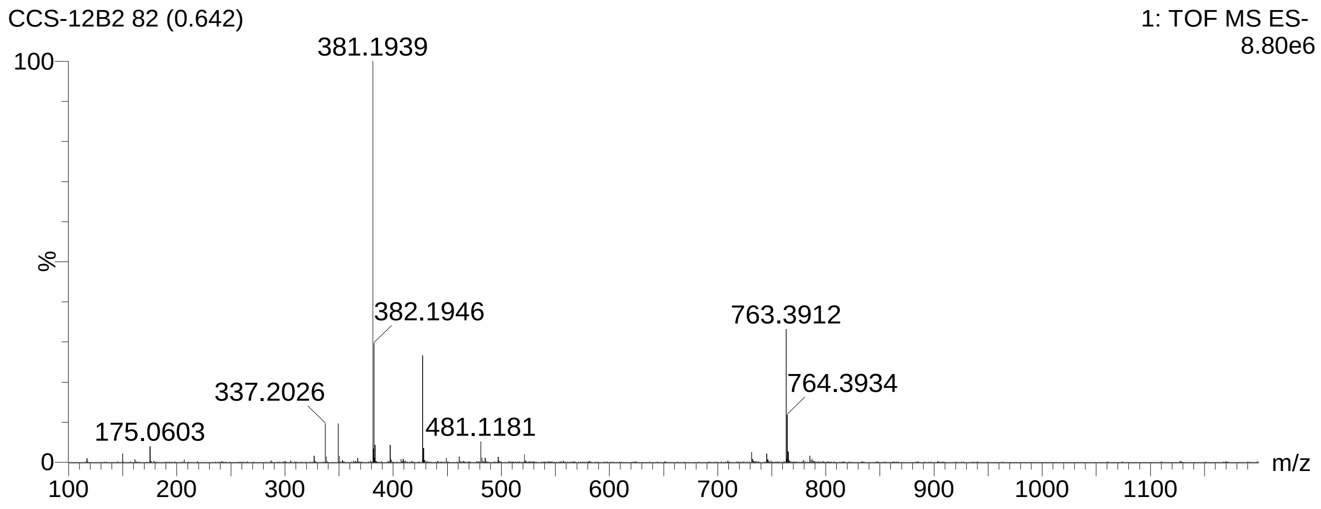

Figure S12 HR-ESI-MS of compound **6**

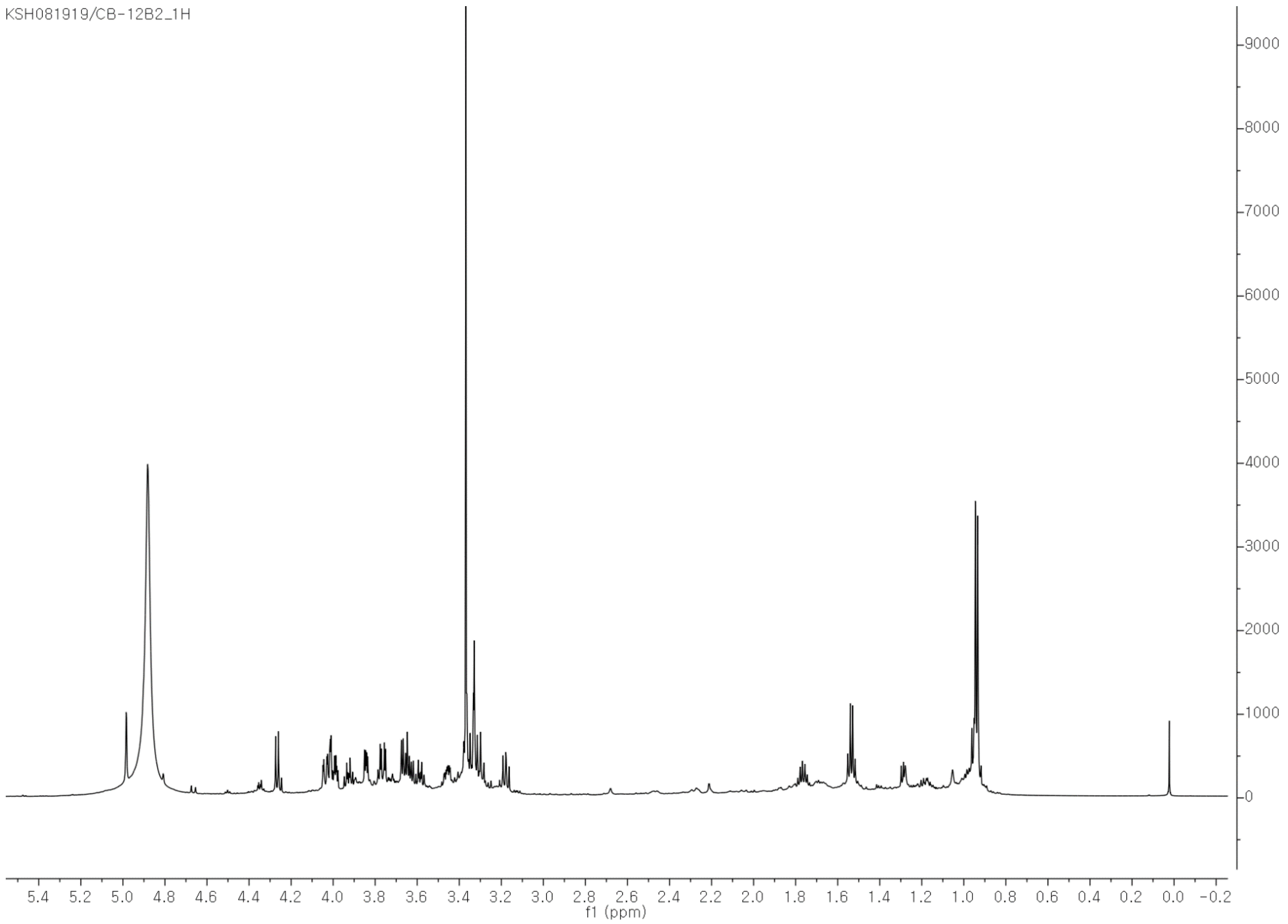

Figure S13 ^{1}H-NMR spectrum of compound **6** (600 MHz, methanol-d_4)

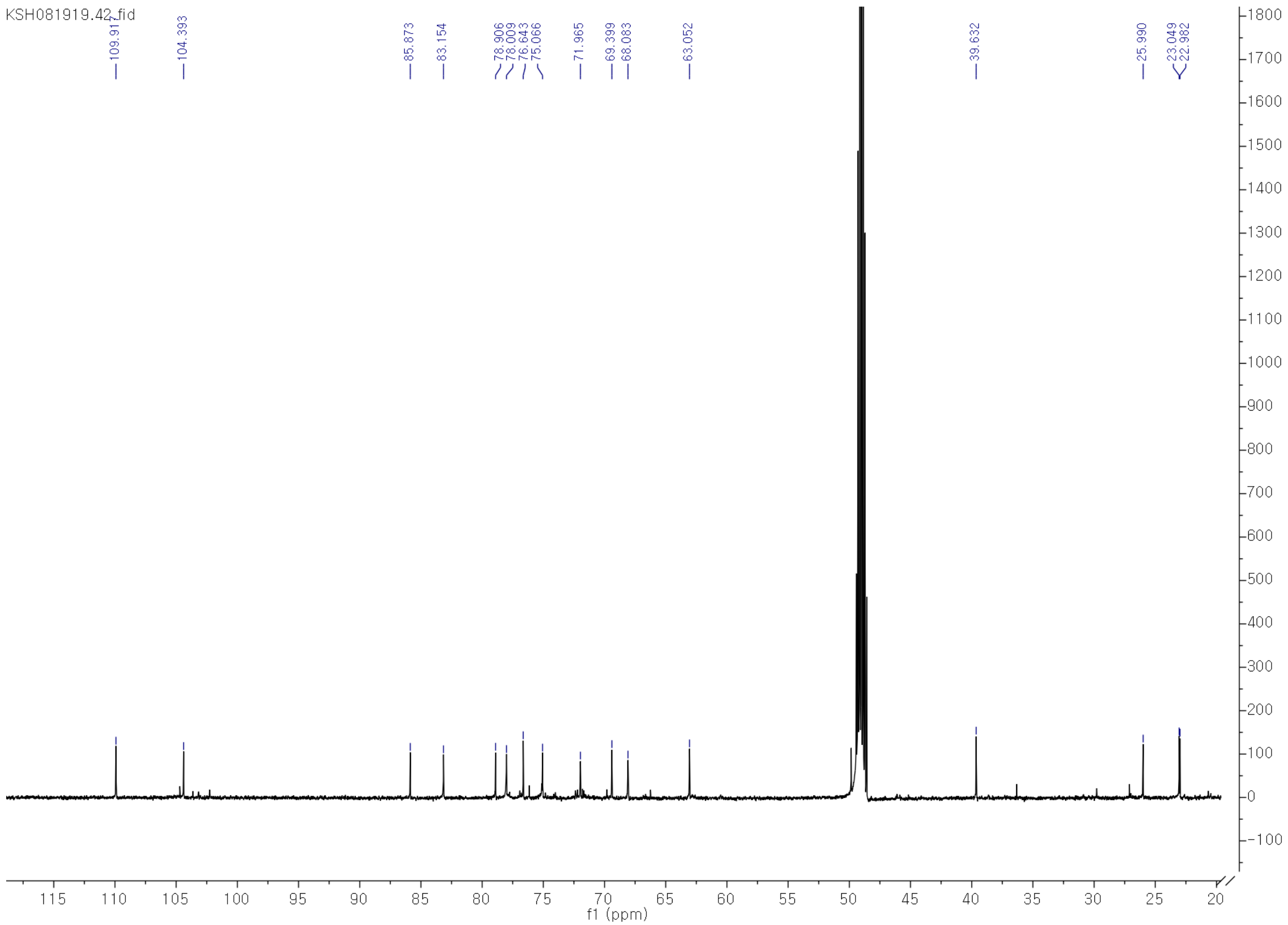

Figure S14 ^{13}C-NMR spectrum of compound **6** (150 MHz, methanol-d_4)

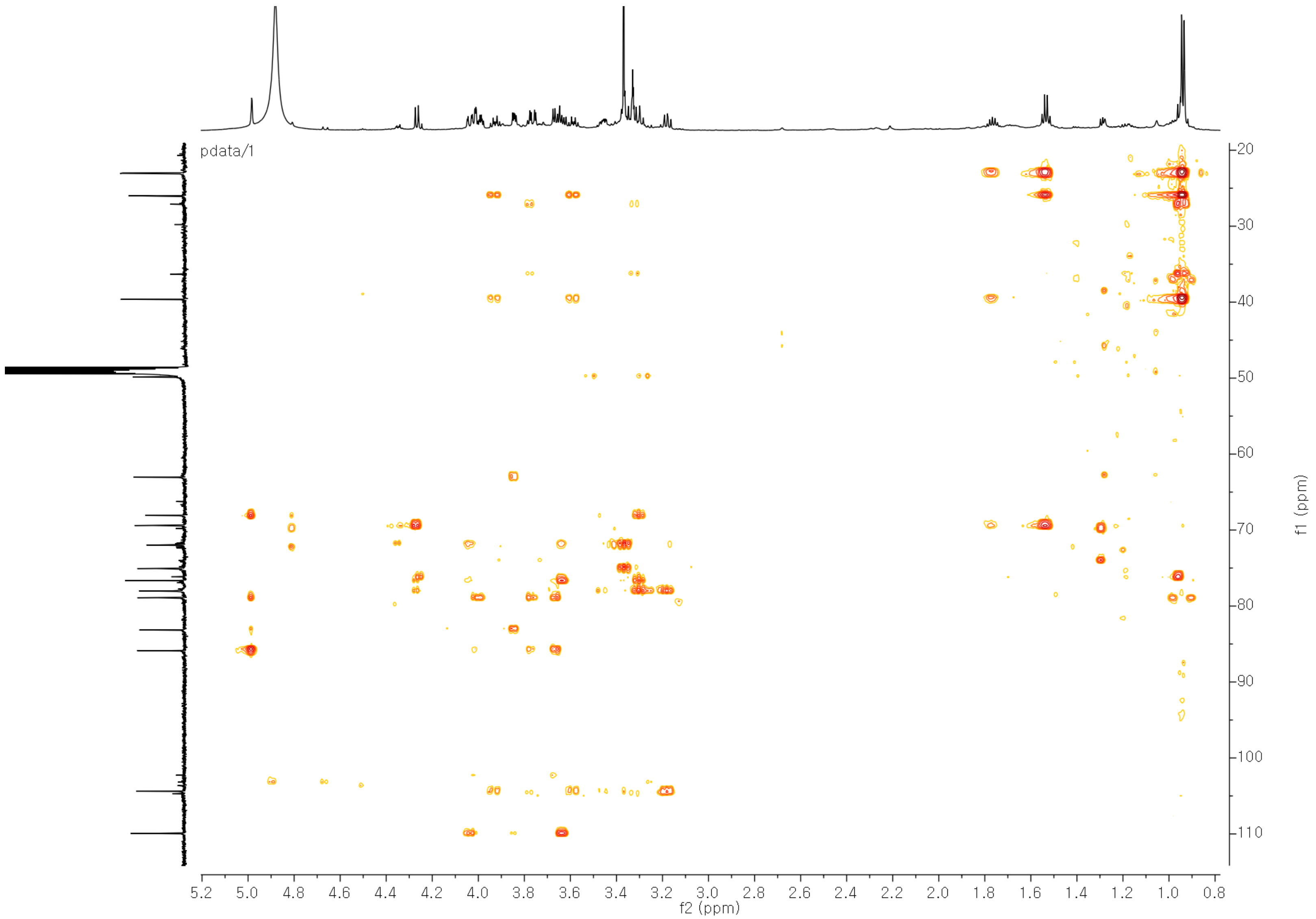

Figure S15 HMBC spectrum of compound **6** (methanol-d_4)

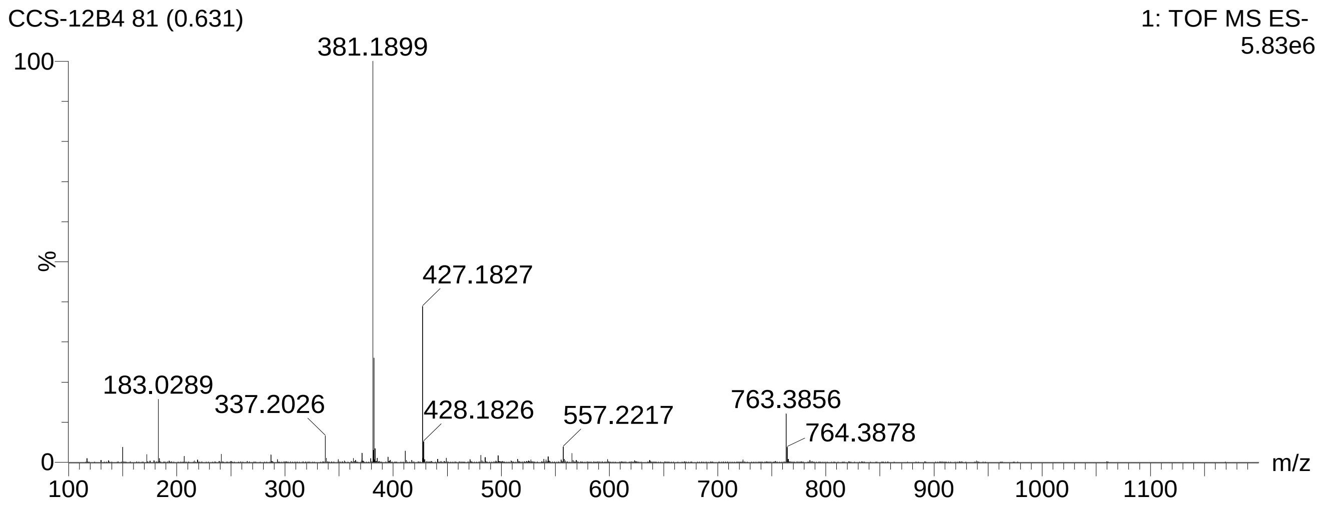

Figure S16 HR-ESI-MS of compound **7**

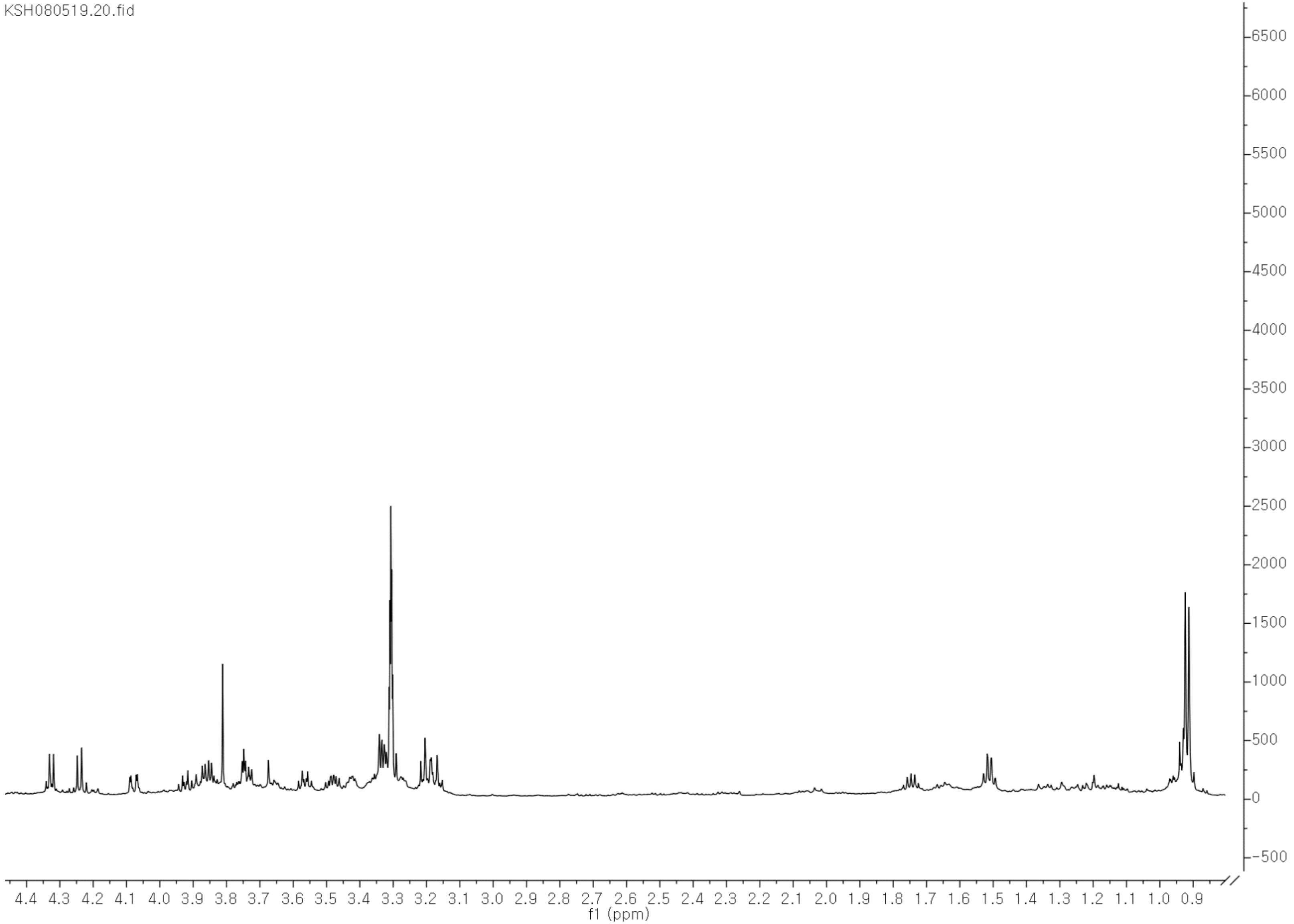

Figure S17 ^{1}H-NMR spectrum of compound **7** (600 MHz, methanol-*d*$_4$)

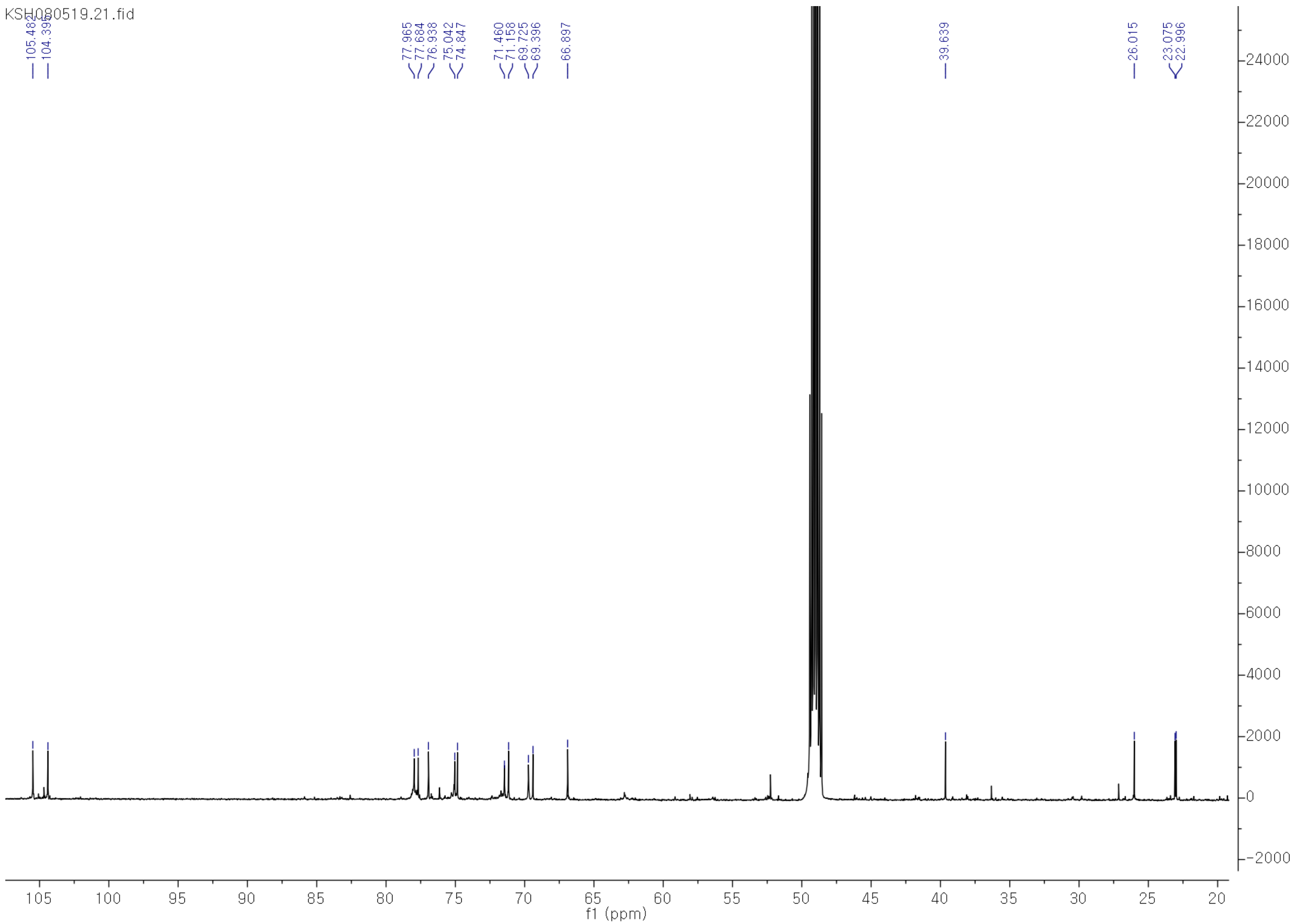

Figure S18 ^{13}C-NMR spectrum of compound **7** (150 MHz, methanol-*d*$_4$)

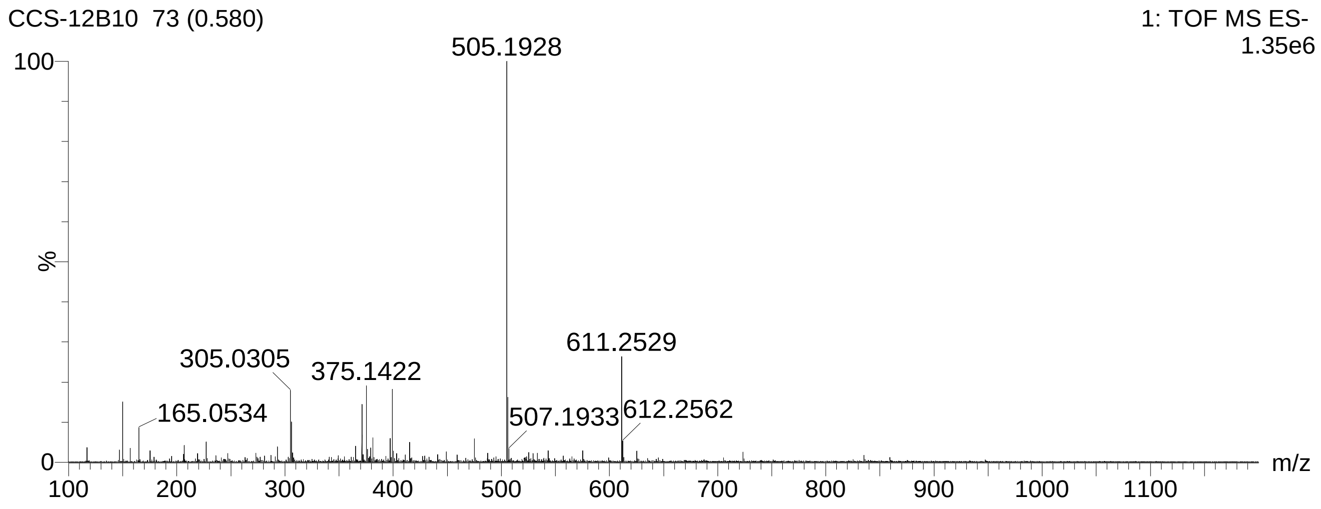

Figure S19 HR-ESI-MS of compound **8**

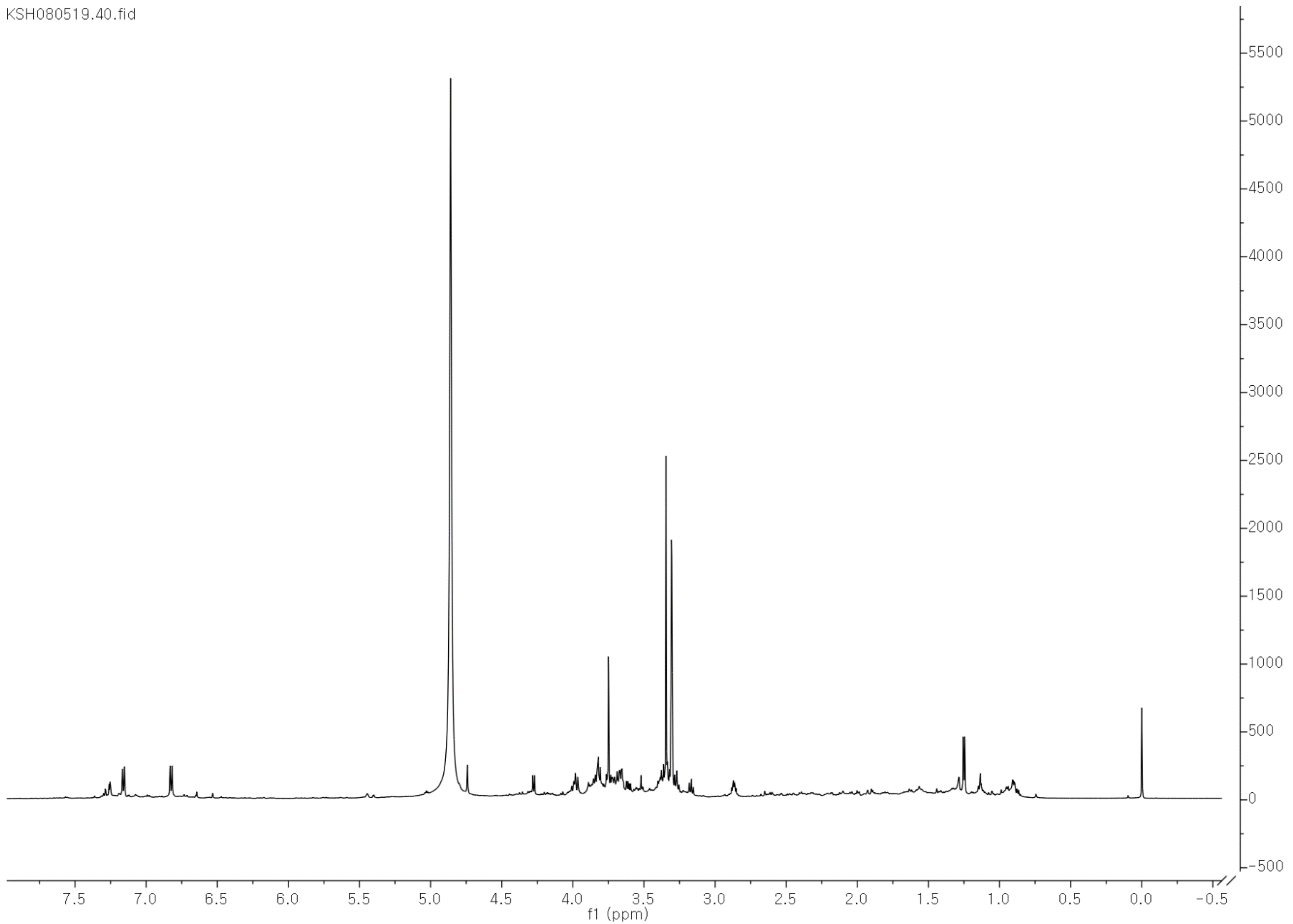

Figure S20 ^{1}H-NMR spectrum of compound **8** (600 MHz, methanol-d_4)

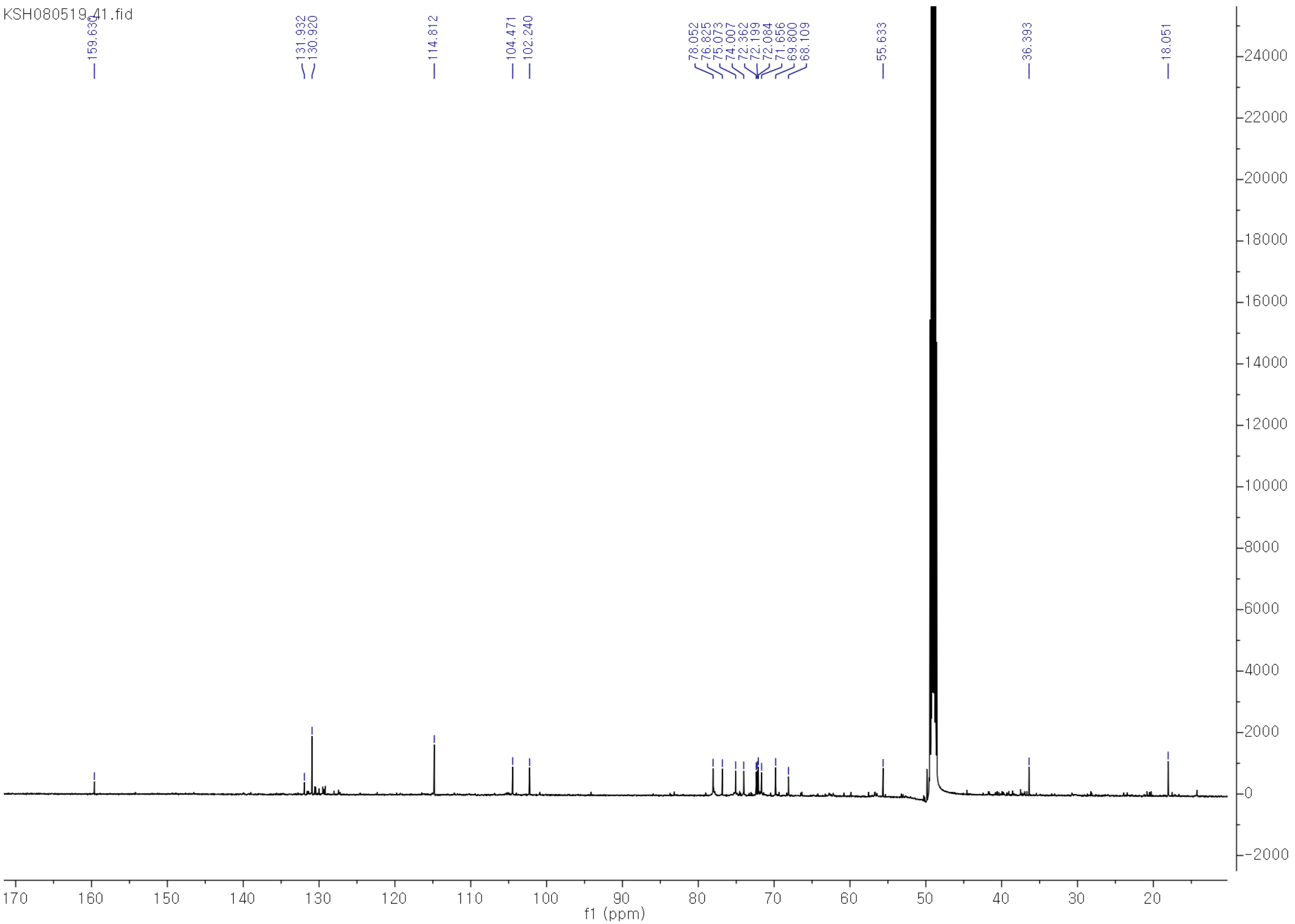

Figure S21 ^{13}C-NMR spectrum of compound **8** (150 MHz, methanol-*d*$_4$)

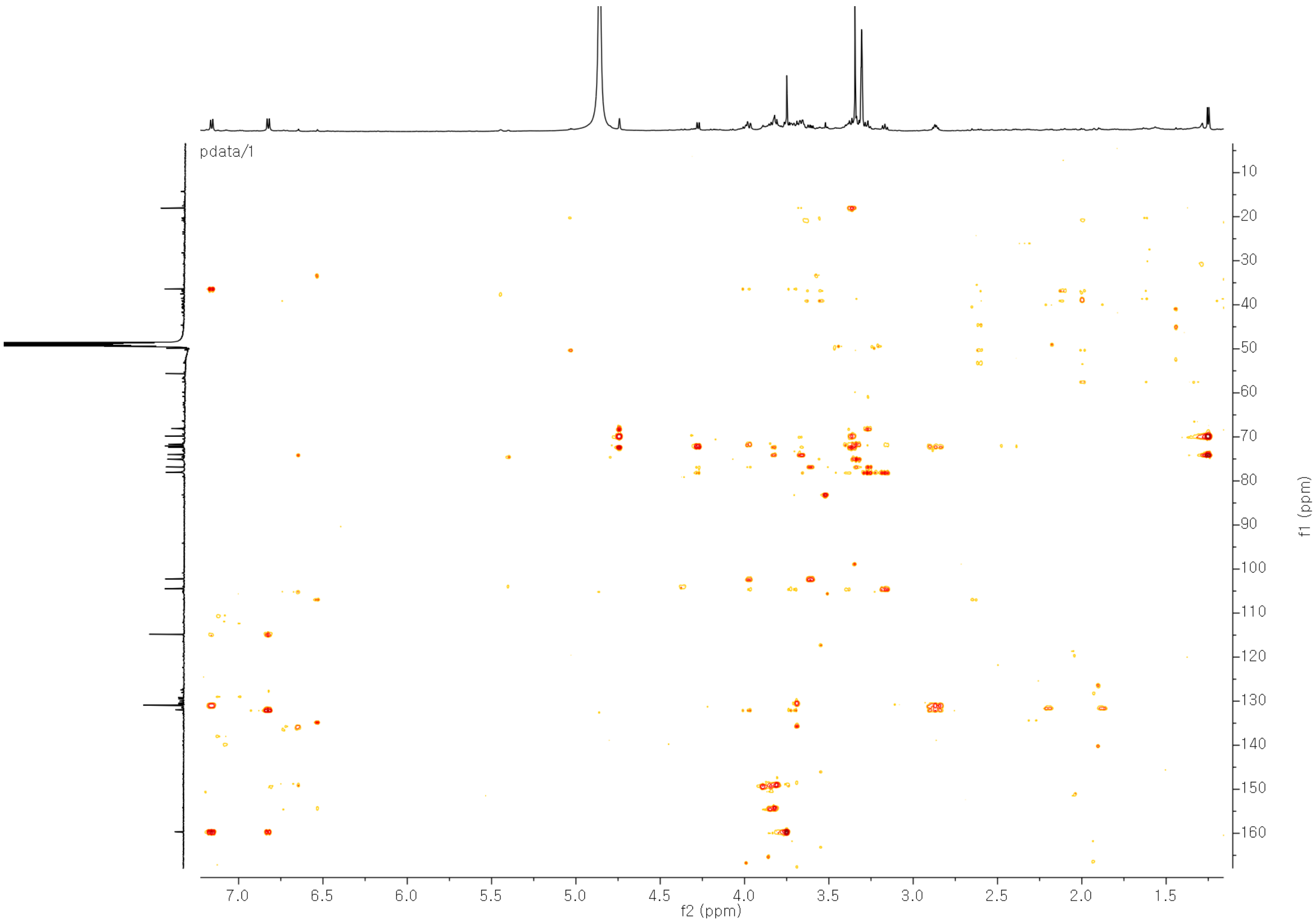

Figure S22 HMBC spectrum of compound **8** (methanol-d_4)
